# Oxygen Reduction Reaction at Single Entity Multiwalled Carbon Nanotubes

**DOI:** 10.1021/acs.jpclett.2c00871

**Published:** 2022-04-21

**Authors:** Yuanyuan Lu, Xiuting Li, Richard G. Compton

**Affiliations:** † Department of Chemistry, Physical and Theoretical Chemistry Laboratory, Oxford University, South Parks Road, Oxford OX1 3QZ, Great Britain; ‡ Institute for Advanced Study, Shenzhen University, Shenzhen, Guangdong 518060, China

## Abstract

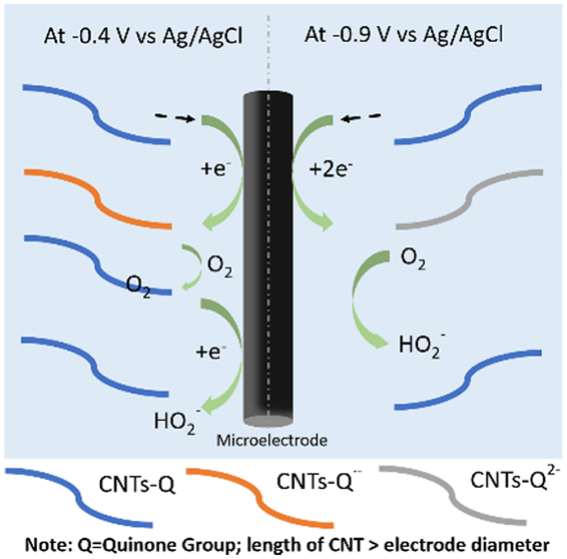

The electrocatalysis of the oxygen reduction reaction (ORR) in aqueous base (0.1 M KOH) by multiwalled carbon nanotubes (MWCNTs) is studied at the single entity level. Electroactive surface functionality is shown to facilitate significant electrocatalysis leading to peroxide formation which is seen to occur at lower potentials as compared to the voltammetric responses obtained at bare carbon macroelectrodes and at such electrodes modified with layers of carbon nanotubes.

Rapid advances in fuel cells facilitate renewable energy generation and give optimism for a sustainable future.^1^ As a core cathodic reaction in fuel cell technology, the oxygen reduction reaction (ORR) has been extensively investigated in the field of electrocatalysis.^2^ Depending on the electrode material, the ORR is found to occur through one of two pathways in alkaline solutions:^3^ a direct four-electron pathway from oxygen to hydroxyl ion (OH^–^) or a stepwise pathway that involves oxygen reduced to hydrogen peroxide anion (HO_2_^–^) first and then followed by further reduction to OH^–^ as the final product. On the basis of voltammetric studies, it has been concluded that the ORR at bulk carbon electrodes predominantly proceeds via the formation of hydrogen peroxide and the initial possible formation of a superoxide anion intermediate indirectly inferred ()^4^ although peroxide is thought to be the likely product in aqueous base.^5^

Multiwalled carbon nanotubes (MWCNTs) have attracted consider attention as possible electrocatalysts for the ORR reaction in both acid^6,7^ and alkaline^8^ conditions, encouraged by the high surface area and the possibility of a multiplicity of active sites^9^ including edge-plane-like defects and oxygen functionality such as quinones where the latter are particularly suggested to play a role at high pH.^10,11^ Such investigations rely on voltammetry and hence the study of layers of carbon nanotubes immobilized on a substrate electrode. As such, the complexity of the mass transport of reactants, intermediates, and products can mask the intrinsic electrocatalytic response of single nanotubes. In the following, we compare the electrocatalytic behavior of MWCNTs for the ORR in aqueous base using ensemble and single entity electrochemistry techniques.^12^ The study of electrochemistry at single carbon nanotubes has recently been shown to be possible^13^ via collisions of MWCNTs at the individual nanotube level by using carbon microelectrodes, where random collisions of the CNTs suspended in aqueous solution leads to transient electrical contact between the electrode and the impacting CNT for periods of up to tens of seconds in favorable cases facilitating electrochemical measurement, in the present case, of dissolved oxygen. If the CNT has greater electrocatalytic character than the impacted microelectrode, this is revealed via currents flowing at potentials which are inert except for the duration of the impact and are clearly signaled by current steps corresponding to the arrival and departure of the CNT from the electrode surface. The experimental details are provided in the Supporting Information (SI Section 1).

Voltammetry was first performed to study the cyclic voltammetry (CV) behavior of MWCNTs modified glassy carbon macro electrodes (MWCNTs/GCE) in 0.1 M KOH under nitrogen degassed conditions. The MWCNT layer on the electrode surface was estimated to be at least ca. 0.7 layers (see SI Section 2), but probably much more from drop-casting 0.1 μg of MWCNTs onto a freshly polished GCE. As shown in Figure 1A, for a sweep potential from 0 to −1.0 V vs Ag/AgCl, no voltammetric signal was seen at the bare GCE, but a well-defined reductive peak at −0.35 V was observed at the MWCNTs/GCE (red curve). The reductive peak current had a linear scan rate dependence as shown in Figure 1B and also increased linearly with the amount of MWCNTs dropcast (Figure S3), which was attributed to the reduction of surface-bound oxygen-containing groups (for example, quinone-like groups) from MWCNTs as reported in the literature.^6,14^ By holding the electrode potential at 0.5 V for a period of minutes with a previous scan from 0 to 0.5 V, the reductive signals were reduced or eliminated after 5 min (see Figure S4), suggesting that the quinone groups on the surface of MWCNTs may be irreversibly oxidized to carboxylic or other groups.^15^ The oxidation may be facilitated by the presence of the high concentrations for hydroxide ions since, at least in the case of benzoquinone in solution, these form an adduct when the pH is above ca. 12:^16^
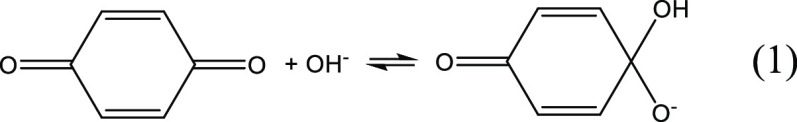
1Last, we note that a single peak is observed in the reductive voltammetry of the MWCNTs, and this is chemically irreversible; the reductive peak is absent in subsequent scans if multiple CV cycles in the potential range of Figure 1A are made (see Figure S5) as will be discussed further below.

**Figure 1:**
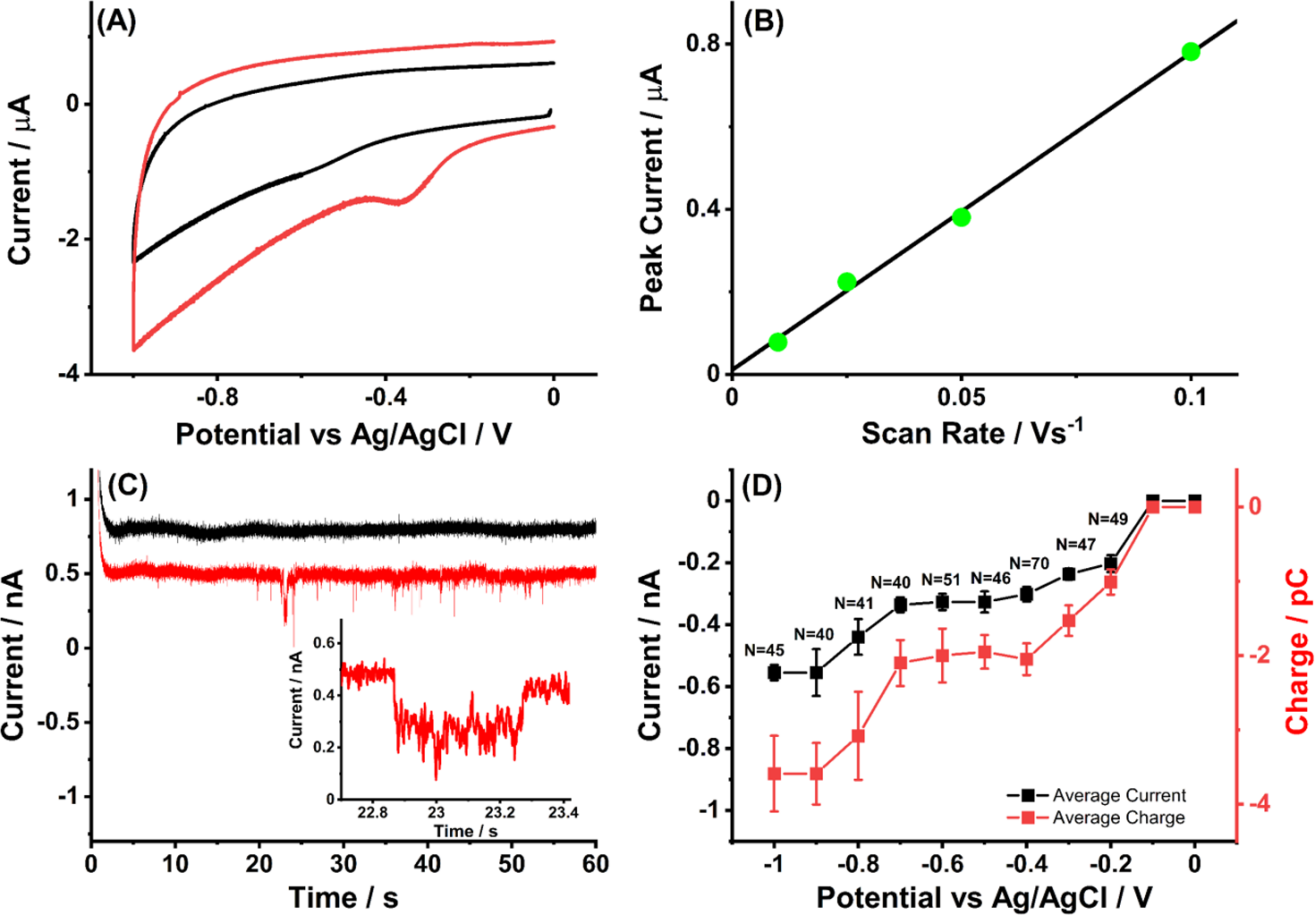
(A) Comparison of cyclic voltammograms measured at a bare GCE (black line) of surface area 7.1 × 10^–2^ cm^2^ and the same GCE modified with 0.1 μg of MWCNTs (red line) in the potential window from 0 to −1.0 V at a scan rate of 50 mV s^–1^. (B) Plot of reductive peak current observed at a MWCNTs/GCE in the presence of oxygen (red line in A) versus scan rate from 10 mV s^–1^ to 25, 50, and 100 mV s^–1^ after baseline subtraction as shown in Figure S2. (C) Chronoamperometric profiles showing reductive single CNT impact signals at −0.4 V vs Ag/AgCl (C) vs Ag/AgCl in the presence (black line) or absence of 0.001 g L^–1^MWCNTs (red line). The inset shows enlargements of individual impact. (D) Plot of average impact current (black dots) and impact average charge (red dots) as a function of potential from 0 to −1.0 V (the error bars represent the average of at least 40 separate impacts at a carbon microwire electrode for each potential). All the experiments were conducted in nitrogen degassed 0.1 M KOH.

Nano impact experiments were conducted to further probe the redox behavior of MWCNTs surface functionality via the collision of individual MWCNTs particles with a carbon fiber microwire electrode in a N_2_-saturated solution containing 0.1 M KOH and 0.001 g L^–1^ MWCNTs. Chronoamperometric scans were made at variable potentials in the range 0 to −1.0 V, giving clear reductive impacts as shown in Figure 1C for the example case of −0.4 V. The average impact duration was 22 ± 2 ms with individual impacts ranging from 11 to 78 ms (at least 40 samples for each potential; see Figure S7A). The frequency of reductive impact responses maintained a value of 0.068 ± 0.011 s^–1^ over the potential range from −1.0 to −0.2 V (Figure S8A),and the potential dependency of the average current and charge is shown in Figure 1D. It shows two current plateaus: the first plateau has an average current height of ca. −0.32 ± 0.03 nA with an average of ca. 1.9 ± 0.3 pC passed when the potential is between −0.4 and −0.7 V, and the second appeared at potentials negative than −0.9 V with ca. −0.55 ± 0.03 nA with an average of ca. 3.6 ± 0.5 pC passed. The half-wave potentials of the two current–potential waves in Figure 1D are ca. −0.20 ± 0.05 and −0.80 ± 0.05 V vs Ag/AgCl, respectively. No reductive impacts were observed for potentials more positive than −0.1 V or in the absence of CNTs. The magnitude of the charge passed in the reductive impacts is consistent with 3.2 × 10^14^ molecules/cm^2^ of CNT external surface assuming a two-electron process (SI Section 4), which corresponds to an unrealistically high fraction (1 in 6 for quinone groups to C_6_ hexagons on the tube surface), suggesting the possibility that some aggregates of MWCNTs are involved in the collision process. It is interesting that two features are seen in the single entity current–voltage response whereas just one appears in the CV recorded for the drop-casted GCE. The first single entity “wave” occurs at a similar potential seen for the single feature in the CV, while the absence of a second wave in the latter is consistent with the inferred chemical irreversibility of the former so that the surface feature has been consumed at higher potentials. The observation of two peaks of similar magnitude in the impact responses is again possibly suggestive of the presence of quinones since in aqueous solution a single two-electron CV wave for ensembles is typically seen in the accessible pH range^17^ including in strong alkaline media.^10,18^ While in the hydrophobic environments of nonaqueous solutions, two voltammetric features are seen corresponding to the generation of surface semiquinones (Q^•–^) and dianions (Q^2–^) in separate one-electron processes:^19^
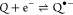
2
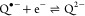
3where Q denotes a quinone group on the CNT. The surfaces of the CNTs are thought, as with basal plane graphite, to have a hydrophobic nature,^20^ so we accordingly suggest the two features seen possibly have their origin in the two one-electron reductions of surface quinones. Anodic impacts were observed at positive potentials as reported in SI Section 5. Last, we note that the shape of individual impact transient has been used to infer the possible collision dynamics in the case of graphene nanoplatelets.^21^ This may also be possible in the case of MWCNTs but is beyond the scope of this Letter.

Next, we explored the ORR at MWCNTs. For comparison, cyclic voltammograms were scanned at a freshly polished GCE and MWCNTs/GCE in 0.1 M KOH in the absence and presence of O_2_ over the potential range 0 to −1.4 V; no signals were seen in the absence of oxygen (Figure 2A). The dominant features seen in the bare GCE voltammograms are the peak at −0.56 V (Peak_1_) and a much smaller peak at −0.76 V (Peak_2_), as shown in Figure 2A. Voltammetric analysis of the two peaks is described in SI Section 6. Note that both peaks are associated with overall reduction to peroxide as fully discussed in previous literature,^5^ where the role of surface-adsorbed species is identified in bringing about a two-electron reduction at high pH, in contrast to the solution phase only mechanism at pH < 10 where, counter to thermodynamic expectations, the reduction appears at less cathodic potentials at high pH (0.1 M KOH). Note that the reported quasi-reversible formal potential for the O_2_/ couple is −0.39 V vs Ag/AgCl,^22^ so that an overall simple one-electron process can be discounted. The literature^5^ indicates that the likely surface chemistry involves the formation of adsorbed hydroperoxide species at the electrode surface with subsequent loss of peroxide ions, , in basic but not under neutral conditions where the hydroperoxide is protonated and remains on the surface.

**Figure 2:**
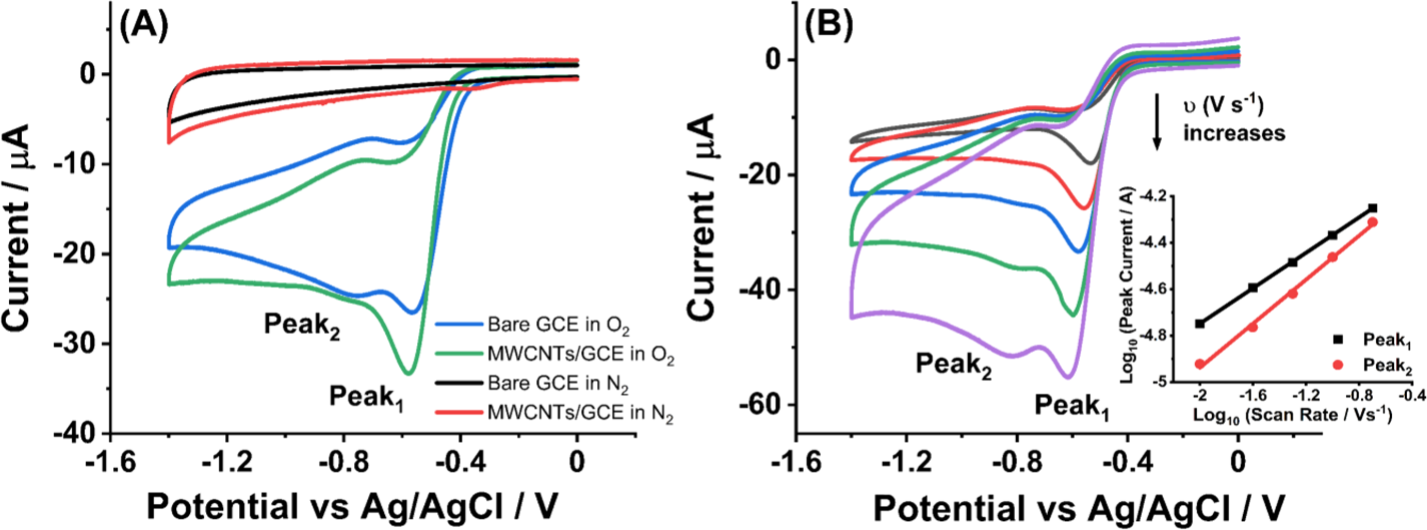
(A) Voltammograms of bare GCE and the same GCE modified with 0.1 μg of MWCNTs in 0.1 M KOH in the presence or absence of O_2_ at a scan rate of 50 mV s^–1^. (B) Voltammograms at the 0.1 μg MWCNTs modified GCE in 0.1 M KOH with saturated O_2_ as a function of scan rate. Inset: plot of peak currents vs scan rates in a log–log form for Peak_1_ (*R*^2^ = 0.99, slope = 0.38) and Peak_2_ (*R*^2^ = 0.99, slope = 0.47).

Figure 2A also shows the corresponding voltammetry recorded at the same GCE after modification with a drop-casted layer of 0.1 μg of MWCNTs. The corresponding scan rate dependence is shown in Figure 2B, from which the peak potentials of both peaks shift cathodically with scan rate, suggesting some degree of electrochemical irreversibility while the current of Peak_1_ increases relative to Peak_2_. Figure S10 shows that if the amount of MWCNT drop-cast on the surface increases, then Peak_1_ also increases approximately linearly with the coverage, suggesting the role of thin layer effects on the voltammetric response.^23,24^ Comparison of the response of the bare and MWCNTs modified GCE indicated that the currents are slightly higher for both peaks on the MWCNTs modified GCE, but otherwise the voltammograms are rather similar except at higher coverages reflected by the increase in Peak_1_ (Figure S10). The possible causes for differences include changed electrode kinetics between the glassy carbon and the CNTs, altered numbers of sites for adsorption of intermediates, changed diffusion to and from the electrode with the porous layer promoting thin-layer like response as reported in ref (23), and altered homogeneous chemical reactivity of the superoxide and other species within the porous layer.

Having tentatively inferred a *possible* catalytic effect of MWCNTs toward the ORR from ensemble electrochemistry, single entity electrochemistry was then conducted at the single MWCNTs entity level with the anticipation of establishing a clear catalytic effect or otherwise. A carbon fiber microwire electrode was inserted into a suspension of 0.001 g L^–1^ MWCNTs in a 0.1 M KOH solution with saturated O_2_, and then chronoamperograms were recorded at −0.6 V vs Ag/AgCl (red line in Figure 3A; for more examples see Figure S11). Clear reductive current steps were seen while no impact signals were detected in the absence of MWCNTs (black line in Figure 3A). A steady-state ORR reduction wave was observed on the carbon wire electrode in the absence of MWCNTs with a half-wave potential at −0.49 V and a quasi-limiting current at −0.8 μA (Figure S12). The wave is “drawn out”, probably reflecting the merging of the two peaks seen at the GCE. This is discussed further below.

**Figure 3:**
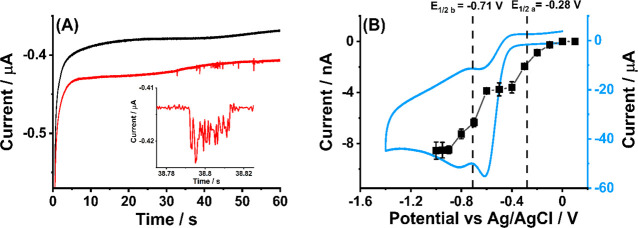
(A) Representative chronoamperometric profiles showing impact signals in oxygen-saturated 0.1 M KOH at −0.6 V vs Ag/AgCl using a carbon wire electrode with presence of 0.001 g L^–1^ MWCNTs (black line) or not (red line) (inset: the enlarged impact signals). (B) Potential dependence of the average impact currents for oxygen reduction reaction catalyzed by MWCNTs using carbon wire, shown for comparison is a CV recorded at a MWCNTs modified GCE for ORR at a scan rate of 200 mV s^–1^. Vertical dashed lines represent the half-wave potential (*E*_1/2_) at each step.

A potential variation study was conducted for the microwire electrode at a series of applied potentials from 0.1 to −1.0 V. Figure 3B shows the average current height of the impact features as a function of potential. Two steps are seen in which the half-wave potentials in the single entity “voltammograms” are −0.28 and −0.71 V. The limiting current of the first wave is 3.7 nA and of the second wave is 8.6 nA. The average residence time was independent of potential with an average value of 68 ± 15 ms (based on at least 30 samples for each potential; see Figure S13). The potential of the first wave is anodic of the reversible oxygen/superoxide couple but occurs at a similar potential to that seen for the reduction of the CNTs in the absence of oxygen. Similarly, the second wave occurs at potentials consistent with those required for the observation of the second wave seen in the absence of oxygen. The much greater currents flowing when oxygen is present revealed by the reductive impacts with similar average frequency (0.060 ± 0.013 s^–1^, Figure S14) indicate that the features suggested above to be related to the two one-electron reductions of surface quinone on the CNTs are responsible for catalytic oxygen reduction (see Figure S15).

Figure S12 shows that impact signals are seen at potentials less negative than required for the ORR at the microwire electrode. The magnitude of the impact signal is ca. 2 orders of magnitude lower than that for the steady ORR signal seen at the microwire electrode with absence of CNTs. The reason that impact signals are also apparent even at high negative potentials and appear superimposed on the underlying ORR current at the microwire electrode is that we suppose the CNT to make electrical contact via its end or side, and because the tube has a larger length than the substrate electrode radius, the CNT extends beyond the diffusion layer near the electrode. Thus, the partial depletion of oxygen at the microwire electrode does not stop impact signals being seen. At the same time distinctive ORR impact signals are seen at potentials where no current flows on the underlying microwire electrode. The diffusion-controlled limiting current for the reduction of oxygen at a single MWCNT tube is estimated to be ∼8 nA, assuming a two-electron process and that the diffusion is to a microcylindrical electrode with a radius of 15 nm and a length of 20 μm (SI Section 12).^25^ Comparison of Figures 1D and 3B suggests that the first step can be ascribed to a kinetically controlled reduction of oxygen by the inferred semiquinone while the doubly reduced quinone mediates a full two-electron electrocatalytic reduction of oxygen under diffusion control. Previous work has shown the reliability of estimating diffusion-controlled currents to single CNTs on this basis. In summary, we suggest the following electrochemistry:first wave:
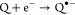
4
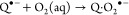
5
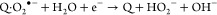
6second wave:
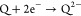
7
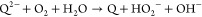
8where the catalysis is sufficiently strong to allow the near diffusion-controlled reduction of oxygen at the single CNTs by the quinone dianions (Q^2–^). This is consistent with earlier speculations^10,26^ about the role of quinones (or other oxygen functionality) in the ORR at carbon electrodes. The magnitude of the current seen for the second wave in the single entity current–voltage plot suggests a two-electron process. It is interesting to reflect on the fact that the reduction of oxygen occurs at a less negative potential at the single nanotubes than seen at the drop-casted CNT electrode as apparent in Figure 3B. Specifically, the question arises as to why oxygen electroreduction is seen at more anodic potentials than for ensembles of nanotubes. The origin likely lies in the fact that in the single entity experiments each point in the current–voltage curve is an average of measurements lasting only tens of milliseconds on fresh entities newly arrived at the electrode surface. In contrast, in the CV experiments, the potential is swept, and the recording of the signal occurs over a period of seconds. As shown above, the surface CV signal inferred to be the first reduction of a quinone leads to the irreversible destruction of the signal; we infer that in CV mode the quinones are destroyed in preference to undergoing electrocatalytic reaction with oxygen.

To conclude, we have shown the role of surface functionality on carbon nanotubes at both the single entity and ensemble level and the conditions under which electrocatalysis takes place characterized.

## Supporting Information Available

Experimental Section, estimation of the number of layers of MWCNTs drop-casted on the GC electrodes, CV of MWCNTs/GCE in the absence of oxygen, estimation of the surface coverage of quinone at a single CNT, nanoimpact experiment of MWCNTs in the absence of oxygen, voltammetry at unmodified carbon electrodes in the presence of oxygen, voltammetric behavior of MWCNTs/GCE with different modification amount, representative impact samples in the presence of MWCNTs, cyclic voltammograms at the carbon microwire electrode toward ORR, average duration time and frequency of impacts in the presence of oxygen as a function of potential, comparison of potential dependence of impacts in the presence and absence of oxygen, and calculation of the transport limited current for single carbon nanotubes (PDF)
Transparent Peer Review report available (PDF)
