# Supplementary material for: Oxygen Reduction Reaction at Single Entity Multiwalled Carbon Nanotubes
Source: J Phys Chem Lett. 2022 Apr 21;13(16):3748–53. doi: 10.1021/acs.jpclett.2c00871 (PMC9059181; doi:10.1021/acs.jpclett.2c00871)
Supplement: Supplementary file 1 — jz2c00871_si_001.pdf [file jz2c00871_si_001.pdf]

-Supporting Information-

The Oxygen Reduction Reaction at Single Entity Multiwalled Carbon Nanotubes

Yuanyuan Lu[1], Xiuting Li[2], and Richard G. Compton[1*]

[1] Department of Chemistry, Physical and Theoretical Chemistry Laboratory, Oxford University, South Parks Road, Oxford OX1 3QZ, Great Britain

[2] Institute for Advanced Study, Shenzhen University, Shenzhen, Guangdong 518060, China

* Corresponding author: Richard G. Compton (Email: richard.compton@chem.ox.ac.uk)

Table of Contents

Section 1: Experimental Section 2

Section 2: Estimation of the number of layers of MWCNTs drop-casted on the GC electrodes 4

Section 3: CV of MWCNTs/GCE in the absence of oxygen 5

Section 4: Estimation of the surface coverage of quinones at a single CNT 7

Section 5: Nano-impact experiment of MWCNTs in the absence of oxygen 8

Section 6: Voltammetry at unmodified carbon electrodes in the presence of oxygen 10

Section 7: Voltammetric behavior of MWCNTs/GCE with different modification amount 11

Section 8: Representative Impact samples in the presence of MWCNTs 12

Section 9: The cyclic voltammograms at the carbon microwire electrode towards ORR 13

Section 10: The average duration time and frequency of impacts in the presence of oxygen as a function of potential 14

Section 11: The comparison of potential dependence of impacts with presence of oxygen or not 15

Section 12: Calculation of the transport limited current for single carbon nanotubes 16

References 17

Section 1: Experimental Section

Chemicals and reagents.

All chemicals were of analytical grade and were used as received without any further purification. Bamboo like multiwall carbon nanotubes (MWCNTs, BPD30L5-20) were purchased from Nanolab ((Brighton, MA, USA). Transmission electron microscopy (TEM) of the sample of MWCNTs is shown in Figure S1 supplied by NanoLab [1]. XPS data analysis [2] of MWCNTs indicated that the sample was 98.9 atomic% carbon, 1.0 atomic% oxygen, 0.1 atomic% iron, and trace (< 0.1 atomic%) copper and sulfur was observed. The dimensions for carbon nanotubes are listed in Table S1.

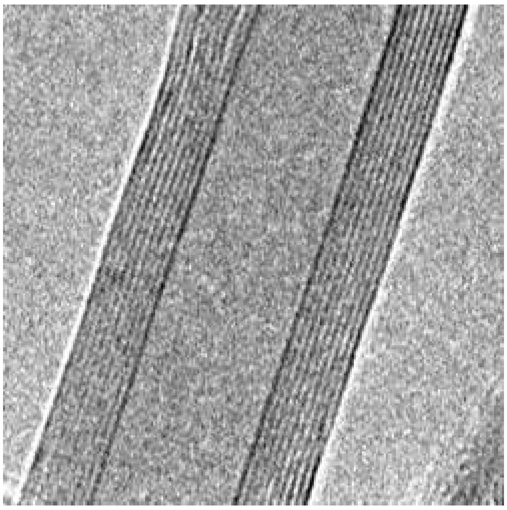

Figure.S1 The transmission electron microscope (TEM) image of a typical single MWCNT from NanoLab [1]

Table S1 The dimensions of MWCNTs

Parameters	
Diameter	30 ± 15 nm
Length	20 μm
External Surface area	1.9 ×10^{-12} m^2
Number of concentric tubes	9

Potassium Hydroxide (KOH, 85%) and Ethanol (≥99.8% purity) were obtained from Sigma-Aldrich (Dorest, UK). All solutions were prepared in ultra-pure water at a resistivity of 18.2 MΩ cm at 298K (Millipore, MA, USA) and were deaerated thoroughly with nitrogen (99.998%, BOC Gases plc) before use.

Electrochemical instrumentation and methods

All electrochemical experiments were conducted in a Faraday cage at 298 ± 0.5 K using an EC-Lab potentiostat (SP-200 with an ultra-low current module, Biologic Science Instruments, France).

For cyclic voltammetry (CV), a glassy carbon macroelectrode (GCE, diameter = 3.02 +/- 0.005mm) was used as the working electrode, a Ag/AgCl electrode (in 3.5 M KCl solution) used as the reference electrode and a graphite rod as the counter electrode. The GCE was polished carefully using alumina with decreasing particle sizes of 1.0, 0.3 and 0.05 μm (Buehler, IL, UK) in turn on soft lapping pads (Buehler, UK), followed by rinsing with ultra-pure water and drying with nitrogen.

For nano-impact experiment, the chronoamperometry was conducted using a carbon fibre micro-wire electrode of 7 μm diameter and 1 mm in length as the working electrode, the same reference electrode and counter electrode were used as above. The fabrication method of carbon fibre micro-wire electrodes has been reported previously [3].

Cyclic voltammetry of oxygen reduction reaction (ORR)

A suspension of 0.05 g L^{-1} MWCNTs was prepared by adding 0.5 mg of MWCNTs to 10 mL ethanol. The suspension was then sonicated for 30 min in a Fisher Scientific FB15050 ultrasonic bath to get a well-dispersed solution. The freshly polished GCE was modified by drop-casting a 2 μL (0.1 μg of MWCNTs) suspension of MWCNTs and left to dry under vacuum.

To investigate possible surface oxygen functional group on the MWCNTs, CVs were conducted using MWCNTs modified GCE in 0.1 M KOH by sweeping the potential from 0 V to –1.0 V under degassed conditions. The voltammograms were recorded at scan rates varing from 10 mVs^{-1} to 25, 50, 100 and 200 mVs^{-1}.

For the cyclic voltammetric behavior of oxygen reduction, the MWCNTs modified GCE were immersed in 0.1 M KOH (measured pH=13.3). Prior to the measurements, the solution was bubbled with pure oxygen gas (O_2, BOC Gases plc, Guildford, UK) for 15 mins. The potential window was chosen to be 0 to -1.4 V and the voltammograms were conducted at different scan rates from 10 to 25, 50, 100 and 200 mVs^{-1}. A control experiment was also performed in the same solution but on a bare GCE.

Nano-impact experiments of MWCNTs

The measurements of current for ORR on single carbon nanotube were based on the general method reported [4] previously. At various potentials in the range from 0.1 V to -1.0 V vs Ag/AgCl, chronoamperometry were performed using carbon micro-wire electrode (diameter = 7 μm) on 10 mL 0.1 M KOH aqueous solution after bubbling with N_2 or O_2 for 15 minutes. Then 0.001 g L^{-1} solution dispersed MWCNTs were added. The data analysis program "Signal Counter" (Centre for Marine and Environmental Research, Zagreb, Croatia) [5] and OriginPro 2020 were employed for impact signal identification and analysis.

Section 2: Estimation of the number of layers of MWCNTs drop-casted on the GC electrodes

To estimate the minimum number of MWCNT monolayers on the bare GC electrode, we assume a closed pack arrangement of the MWCNT is laid uniformly across the whole GC surface. This gives a lower limit estimate for the number of layers.

The diameter and length of the bamboo-like multiwalled carbon nanotubes (purchased from NanoLab, USA) is ca. 30 nm and ca. 20 μm respectively.

Assuming the MWCNTs contact at the GC surface to be rectangular when arranged in a closed pack manner,

The area covered by one MWCNT:

$S_{CNT} = 6 \times 10^{-9}$ cm^2,

The modification amount of MWCNTs (2 μL 0.05 gL^{-1}) on the bare GC electrode:

$m_{CNT} = 1\times10^{-7}$ g

The number of MWCNTs modified on the surface of modified GCE

$N_{MWCNT}=1\times10^{-7}$ g / 1.3×10^{-14} g = 7.7×10^{6} (the mass per carbon tube is 1.3×10^{-14} g [6])

Hence, the total area covered by 7.7×10^{6} MWCNTs

$S_{total} = 6 \times10^{-9}$ cm^2 $\times$ 7.7×10^{6} = 0.046 cm^2

The surface area of GC electrode

$A = (\pi D^2) / 4 = 7.1\times10^{-2}$ cm^2 (the diameter of GC electrode is 3.02 ± 0.005 mm)

The number of monolayers of MWCNTs for 2 μL 0.05 gL^{-1} MWCNTs drop cast

$N_{layer} = \frac{S_{total}}{A}$ = 0.046 cm^2 / 0.07 cm^2 $\approx$ 0.65

Section 3: CV of MWCNTs/GCE in the absence of oxygen

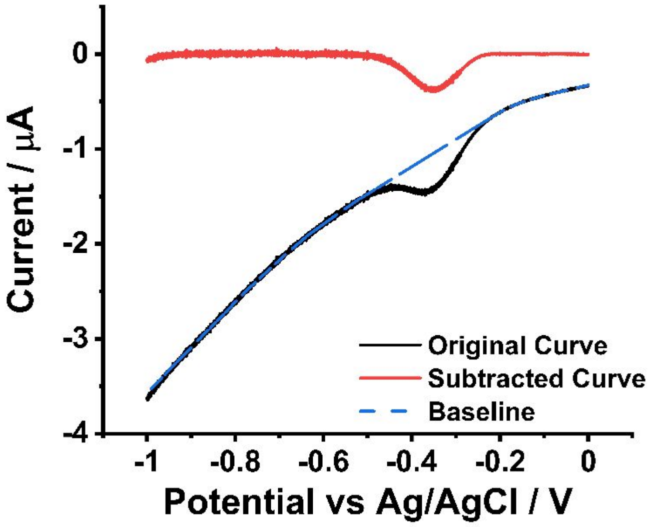

Figure.S2 the baseline subtraction process for cyclic voltammograms of 0.1 μg MWCNTs modified GCE swept forward to -1.0 V at a scan rate of 50 mV s^{-1}.

All cyclic voltammograms of MWCNTs/GCE recorded in the absence of oxygen in 0.1 M KOH were baseline subtracted to obtain the reported values of reductive peak currents as illustrated in Figure S2. The background correction for the raw cyclic voltammograms were conducted by using OriginPro 2020, a baseline was fitted between the potentials at which the surface bound voltammetric wave was judged to have started and ended, then subtracted from the peak.

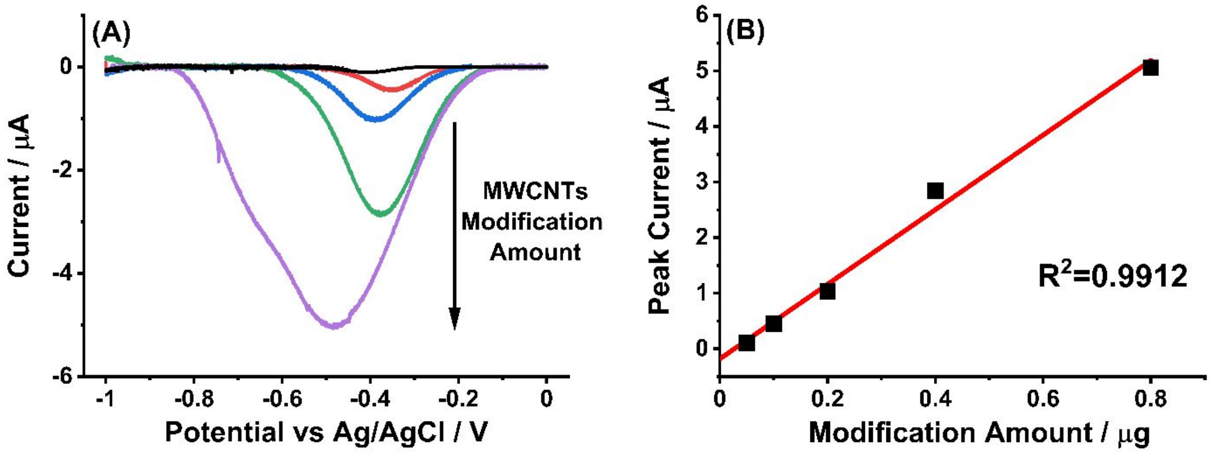

Figure. S3 (A) Cyclic voltammograms of polished GCE in degassed 0.1 M KOH solution with different modification amount of MWCNTs from 0.05 μg to 0.1, 0.2, 0.4, and 0.8 μg swept forward to -1.0 V at a scan rate of 50 mVs^{-1} after baseline correction; (B) The linear relationship between reductive peak current and MWCNTs modification amount.

CVs were recorded at the MWCNTs/GCE in degassed 0.1 M KOH by scanning first from 0 V to 0.5 V and holding the potential at 0.5 V before making the cathodic scan. Under these conditions the reductive peak appearing at -0.35 V decreased in size compared with the CV recorded directly from 0 V to -1 V (Figure S4(A)). Furthermore, as the holding time at + 0.5 V was extended to 1, 3 and 5 mins, the reductive

peak gradually decayed until disappeared (Figure S4(B)). It was inferred that the surface oxygen groups on MWCNTs may be irreversibly converted to carboxylic or other electro-inactive groups under potential control at + 0.5V.

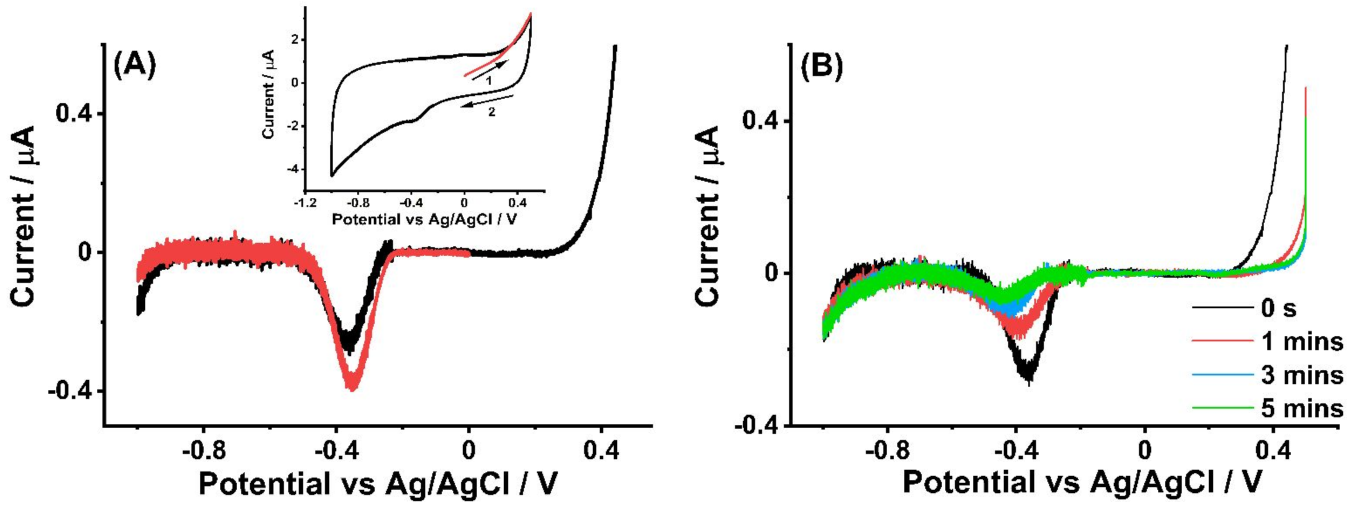

Figure.S4(A) A comparison of cyclic voltammograms measured by 0.1 μg MWCNTs modified GCE in the potential window from 0 V to -1.0 V with a previous scan from 0 V to 0.5 V (black line) or not, inlay: the detailed scan process from 0 V to 0.5 V then back to -1 V; (B) Cyclic voltammograms of 0.1 μg MWCNTs/GCE in the forward scan from 0.5 V to -1.0 V with a previous potential sweep from 0 to 0.5 V then hold at 0.5 V for 0, 1mins, 3 mins and 5 mins. All the voltammograms were at a scan rate of 50 mV s^{-1} and after baseline correction.

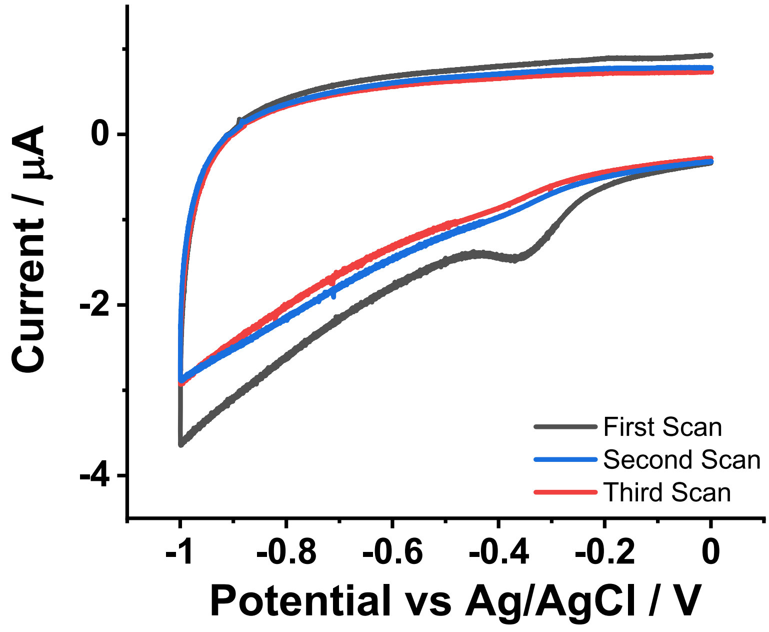

Figure.S5 Cyclic voltammograms of 0.1 μg MWCNTs modified GCE in degassed 0.1 M KOH solution at a scan rate of 50 mVs^{-1} swept from 0 V to -1 V in three successive scans.

Section 4: Estimation of the surface coverage of quinones at a single CNT

Here, we compare the number of quinones with the number of hexagonal C_6 rings on the external surface of the MWCNTs:

The external area (S_{mw}) of one single MWCNT

$S_{mw} = \pi d_{MWCNT} \times L = \pi \times 3 \times 10^{-8}$ m$\times 2.0 \times 10^{-5}$ m$= 1.9 \times 10^{-12}$ m^2,

where d_{MWCNT} (=30 nm) is the diameter of a carbon tube and L (=20 μm) is the length of each tube.

The number of quinone groups (N_q) introduced into a single MWCNTs particle via electro-reduction can be estimated from nano-impact experiments to be

$N_q = Q_{MWCNT\text{-}single}/ne = 1.9 \times 10^{-12}$ C / ($2 \times 1.6 \times 10^{-19}$ C) $= 6 \times 10^6$

where $Q_{MWCNT\text{-}single}$ is the average charge from the reductive spike current when the potential was between -0.4 V and -0.7 V, n is the number of electrons transferred (n=2, assuming a complete two-electron quinone reduction process here).

Therefore, the coverage (Γ) of quinone groups on a single MWCNTs particle is

$\Gamma = N_q / S_{mw} = 6 \times 10^6 / 1.9 \times 10^{-8}$ cm^2 = **3.2×10^{14} molecules per cm^2 of external surface area**

The area (S_h) of one C_6 hexagon composed of six carbon atoms is

$S_h = 3\ d_{c\text{-}c}^2 \times \frac{\sqrt{3}}{2} = 5.2 \times 10^{-20}$ m^2, where $d_{c\text{-}c}$ (= 0.1421 nm) is the length of the C-C bond

The number of hexagons (N_h) on the external surface of a single MWCNTs particle is

$N_h = S_{mw} / S_h = 1.9 \times 10^{-12}$ m^2 / 5.2×10^{-20} m^2 = 3.6×10^7

The amount ratio between quinone groups and hexagons is N_q / N_h = 1:6.

Section 5: Nano-impact experiment of MWCNTs in the absence of oxygen

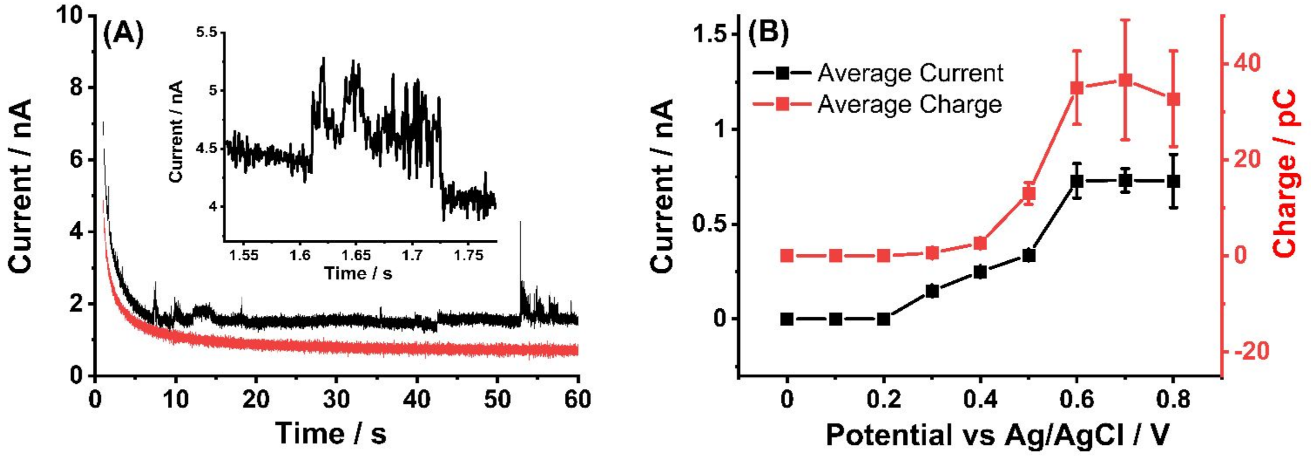

Figure.S6 (A) Chronoamperometric profiles showing oxidative impact signal at 0.5 V vs Ag/AgCl with presence of 0.001 g L^{-1} MWCNTs (black line) or not (red line), the inset is the enlarged impacts; (B) Plot of average impact current (black dots) and impact average charge (red dots) as a function of potential from 0 to 0.8 V. All the experiments were conducted in degassed 0.1M KOH using a carbon fiber microwire electrode.

Single entity measurements were also conducted at oxidizing potentials. Extending the applied potential positive than 0.2 V, oxidative impacts appeared as shown in Figure S6(A) and demonstrated a potential dependency for the impact current height and charge as shown in Figure S6(B). At the more positive potentials the impacts with an average frequency of 0.066 ± 0.015 s^{-1} (Figure S8(B)) show a slightly longer average impact time of length 42 ± 13 ms (see Figure S7(B)). Over the potential range of 0.6 V to 0.8 V, the average current and charge reached a constant level around ca. 0.73 ± 0.09 nA and 35 ± 10 pC. The oxidative impacts are, in the light of the CV experiments mentioned above assigned to the further oxidation of the CNTs, possibly at edge planes and at pre-existing oxygen functionality such as the quinone functionality inferred from the reductive electrochemistry.

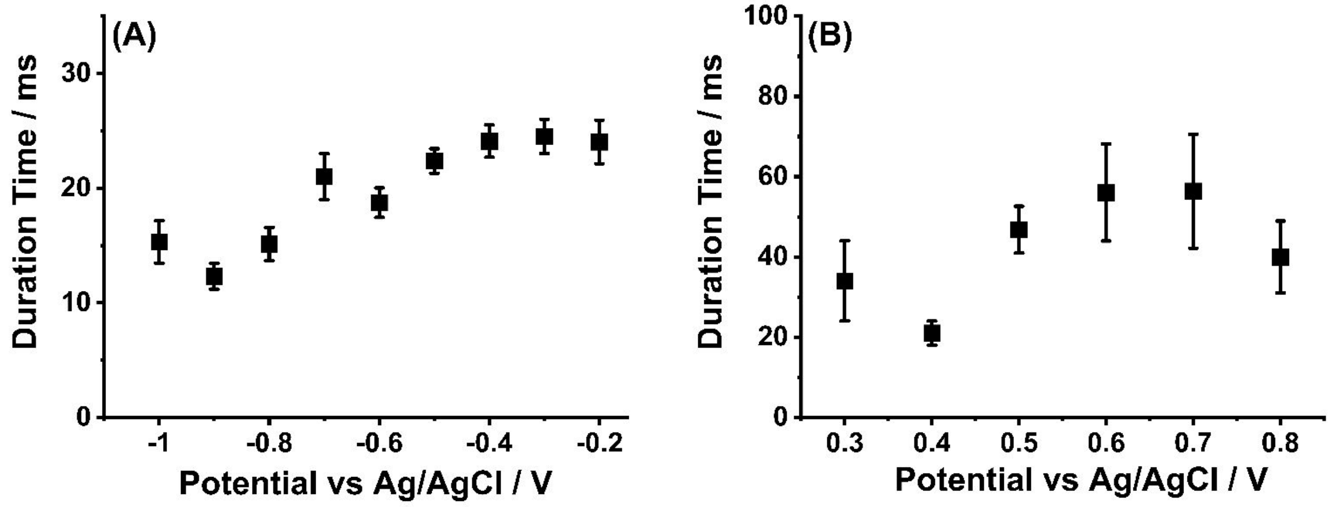

Figure.S7 Plot of average duration time of reductive and oxidative impacts as a function of potential from -1.0 V to -0.2 V (A) and 0.3 V to 0.8 V (B) respectively in the presence of 0.001 g L^{-1} MWCNTs.

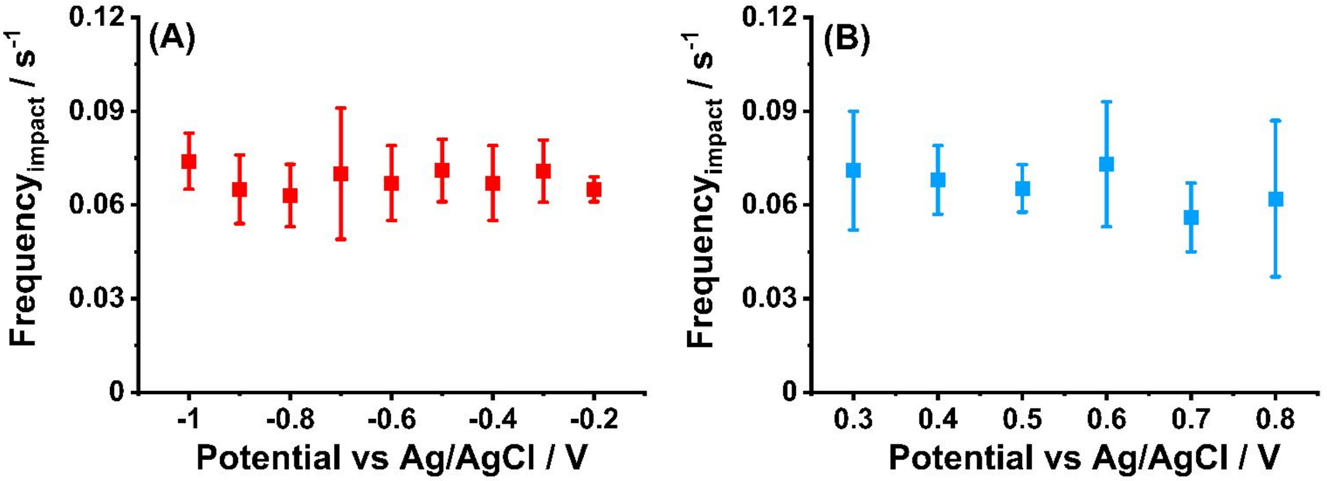

Figure.S8 Impact frequency plot as a function of potential from -0.1 V to -1.0 V (A) and 0.2 V to 0.8 V (B) vs Ag/AgCl in the presence of 0.001 g L^{-1} MWCNTs in nitrogen degassed 0.1 M KOH.

Section 6: Voltammetry at unmodified carbon electrodes in the presence of oxygen

To investigate the ORR performed on a bare GCE in 0.1 M KOH, CVs were scanned for scan rates varying from 10 mVs^{-1} to 200 mVs^{-1} on a bare GCE first from 0 V to -1.4 V vs Ag/AgCl respectively, as shown in Figure S9(A). The inset shows dependence of the peak current on the square root of voltage scan rate hinting at probable diffusion control and the formation of peroxide via a two-electron process as outlined in reference [7]. The Tafel slope is found to vary as a function of scan rate and the calculated cathodic transfer coefficients α_c decreases from 0.79 to 0.48 as scan rate increases (Figure S9(B)) according to Equation S1 defined by the International Union of Pure and Applied Chemistry (IUPAC) [8] as follows:

$$-\frac{RT}{F}\frac{dln|I_c|}{dE} = \alpha^c \qquad \text{Eq.(S1)}$$

where I_c is the experimentally measured cathodic current (in the range of 20 % - 30 %), E is the potential of the working electrode, F is the Faraday constant (96485 C mol^{-1}), R is the Gas Constant (8.314 J mol^{-1} K^{-1}) and T is the experimental temperature (298 K). The interpretation of these data has been given elsewhere [7] where the formation of peroxide species is confirmed and the role of surface adsorbed species is emphasized.

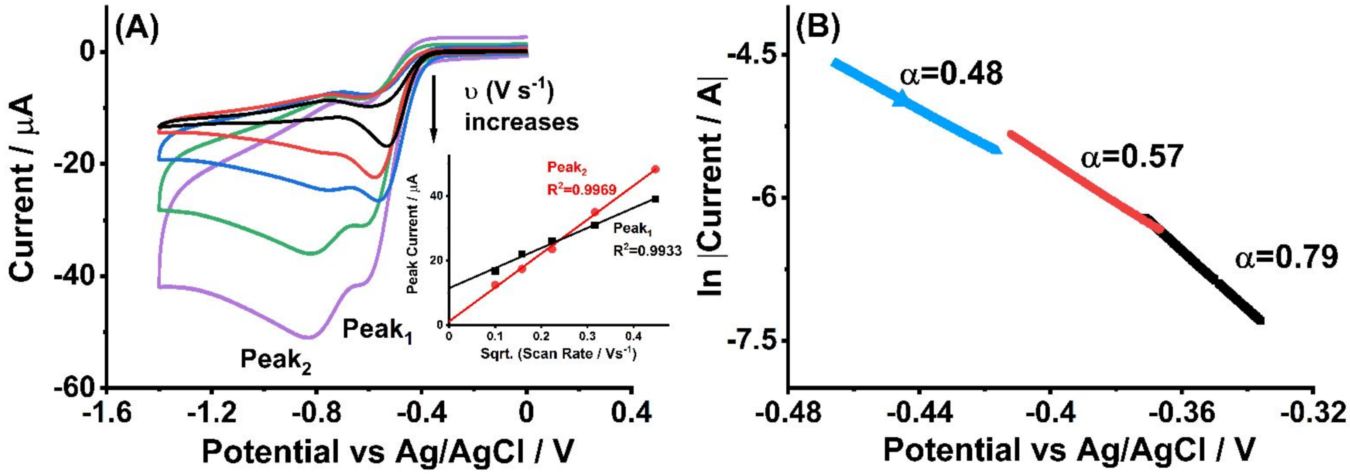

Figure.S9 (A) Voltammograms of bare GCE in oxygen saturated 0.1 M KOH solution at different scan rates from 10 mVs^{-1} to 25, 50, 100 and 200 mVs^{-1} (Inlay: Plot of the peak current-with square root of scan rate for $Peak_1$(black line) and $Peak_2$ (red line)); (B) The Tafel analysis for 10 mVs^{-1} (black), 50 mVs^{-1} (red) and 200 mVs^{-1} (blue).

Section 7: Voltammetric behavior of MWCNTs/GCE with different modification amount

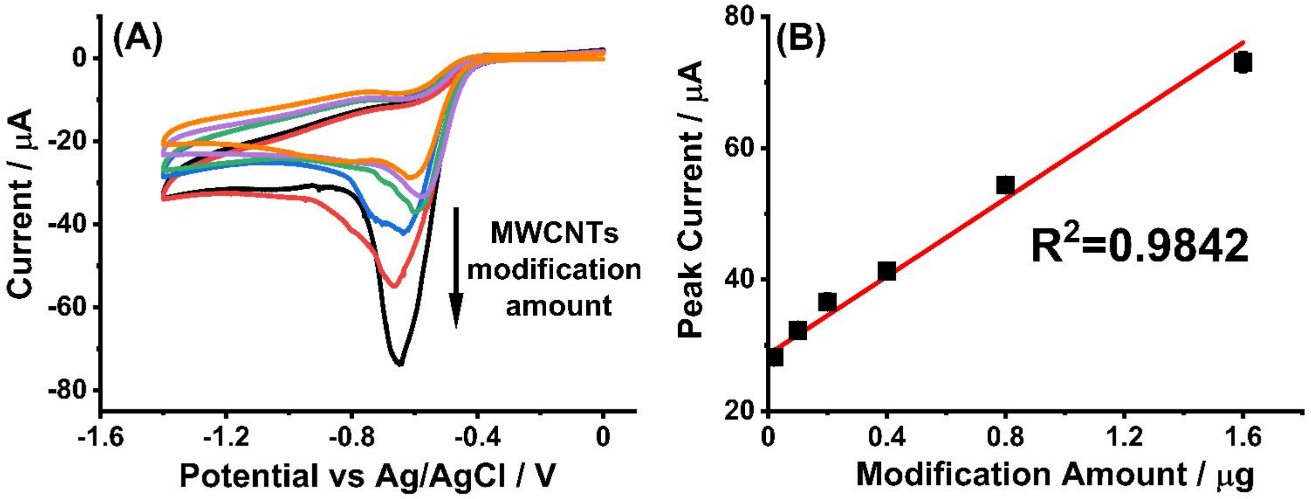

Figure. S10 (A) Cyclic voltammograms of GCE in oxygen saturated 0.1 M KOH solution with different modification amount of MWCNTs from 0.02 to 0.1, 0.2, 0.4, 0.8 and 1.6 μg at a scan rate of 50 mVs^{-1}; (B) The linear relationship between peak current of Peak$_1$ and MWCNTs modification amount.

Section 8: Representative Impact samples in the presence of MWCNTs

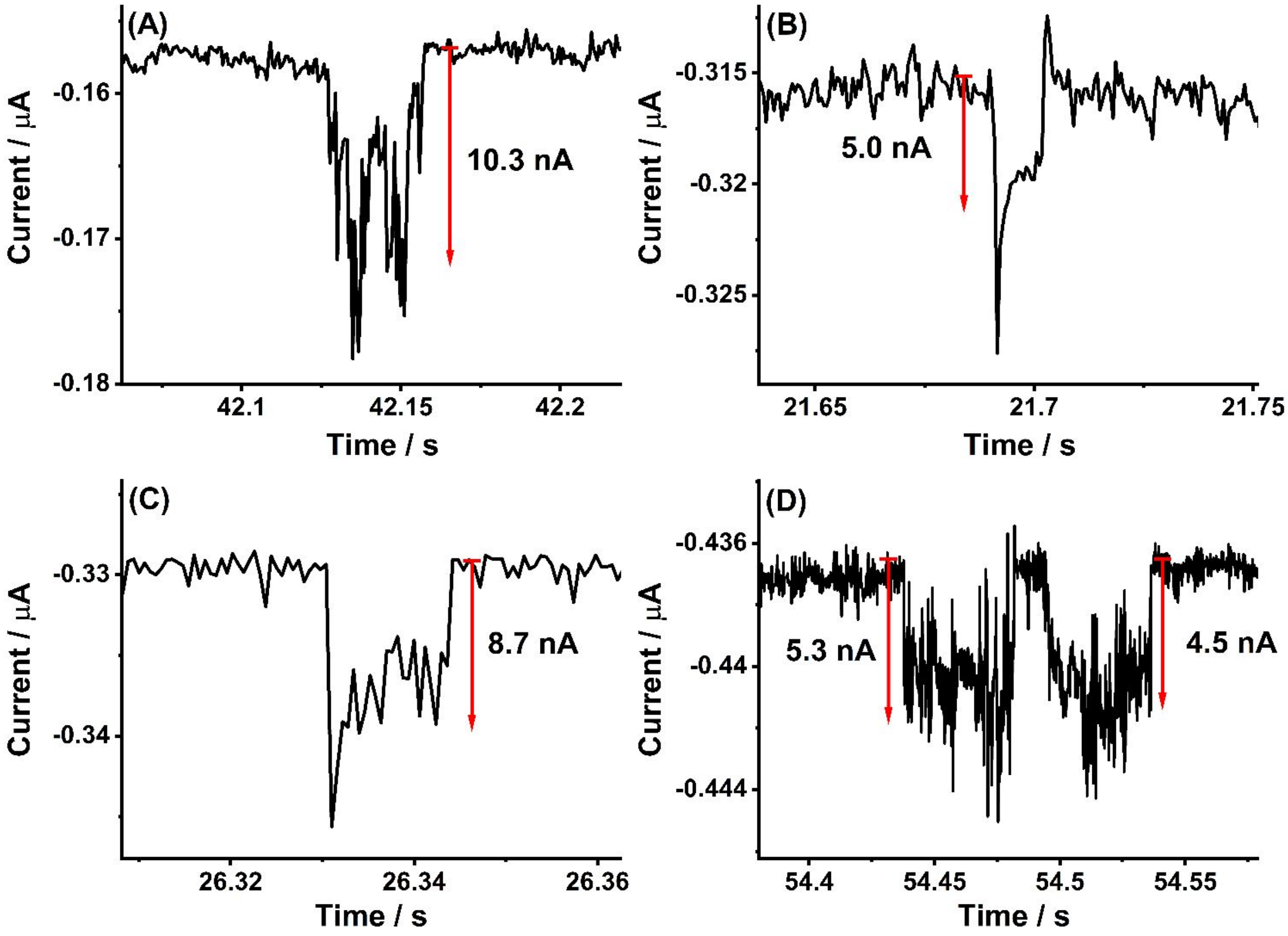

Figure S11 Representative impact signals in oxygen-saturated 0.1 M KOH at -0.6 V vs Ag/AgCl using a carbon wire electrode with presence of 0.001 g L^{-1} MWCNTs.

Section 9: The cyclic voltammograms at the carbon microwire electrode towards ORR

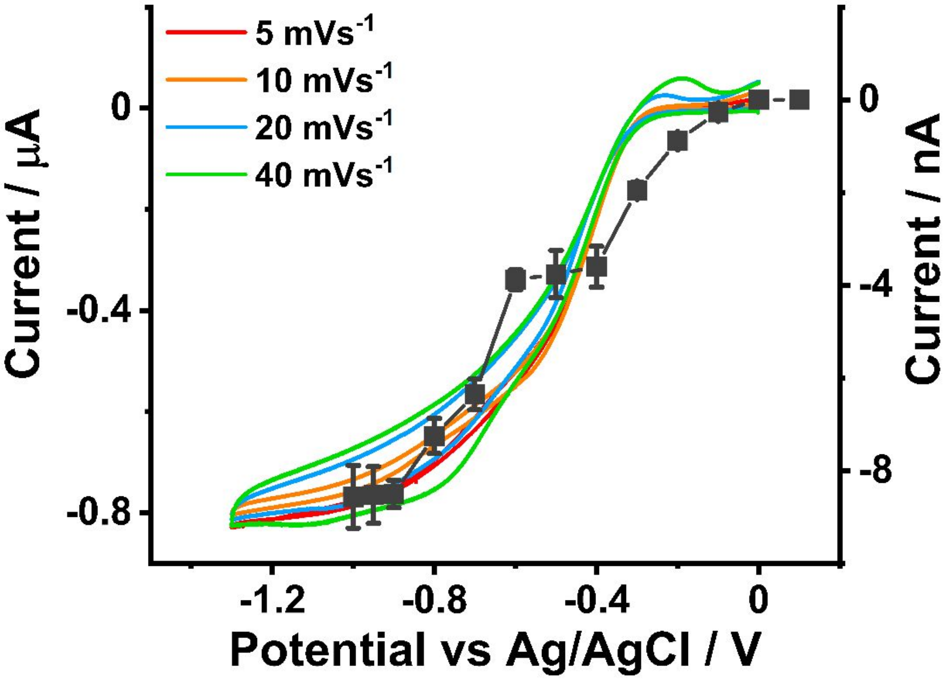

Figure S12 Cyclic voltammograms of carbon microwire electrode in 0.1 M KOH with presence of saturated oxygen in the potential range from 0 V to -1.3 V at different scan rates in comparison with impact currents as a function of potential (orange dots)

Section 10: The average duration time and frequency of impacts in the presence of oxygen as a function of potential

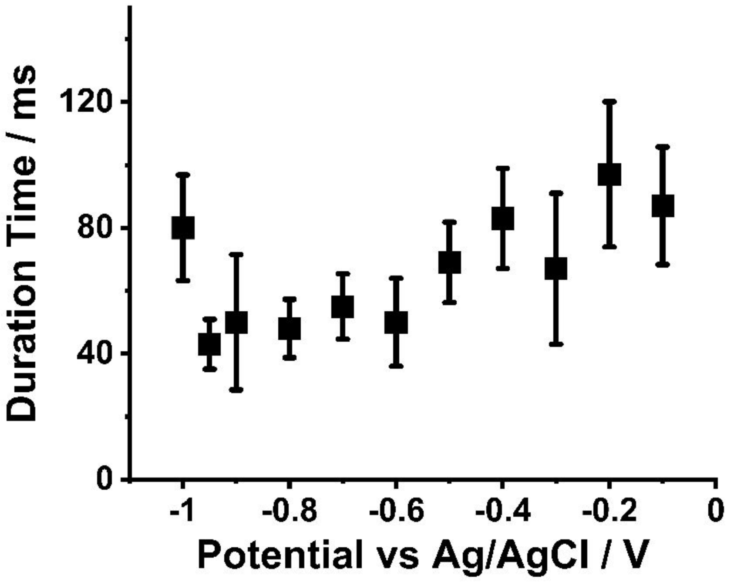

Figure.S13 Impact step average duration time as a function of applied potentials from -0.1 V to -1.0 V vs Ag/AgCl in the presence of 0.001 g L^{-1} MWCNTs.

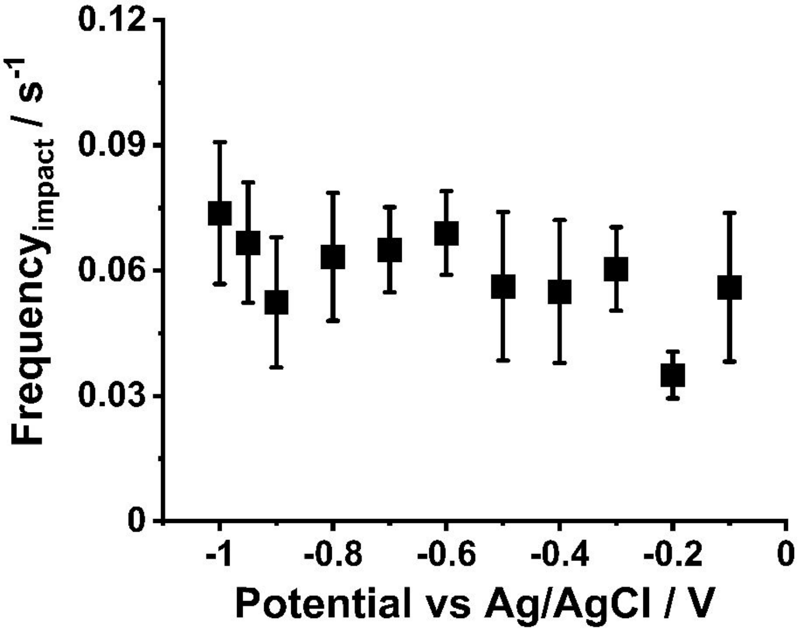

Figure.S14 Impact frequency plot as a function of potential from -0.1 V to -1.0 V vs Ag/AgCl in the presence of 0.001 g L^{-1} MWCNTs in oxygen saturated 0.1 M KOH.

Section 11: The comparison of potential dependence of impacts with presence of oxygen or not

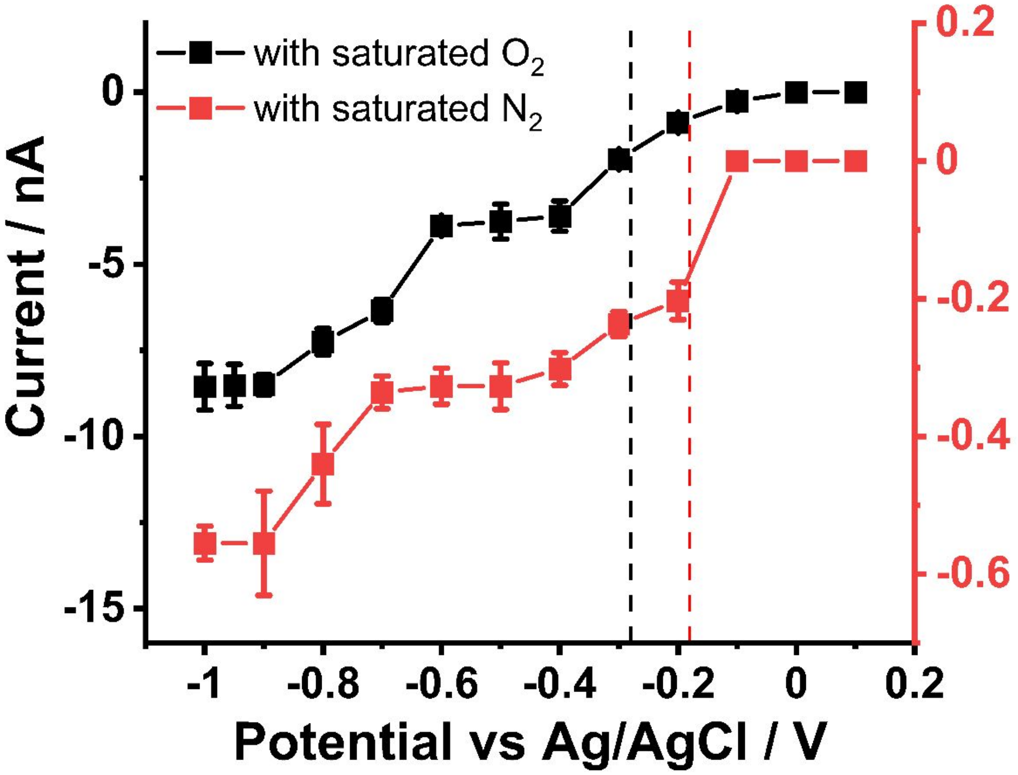

Figure.S15 Potential dependence of the average impact currents in the presence of 0.001 g L^{-1} MWCNTs in a 0.1 M KOH solution with saturated N_2 or O_2. The vertical dashed lines represent the half-wave potential for quinone reduction (red) and oxygen reduction (black) respectively.

Section 12: Calculation of the transport limited current for single carbon nanotubes

To interpret the single nano-imapct events we approximately view the impacted MWCNT as a cylindrical electrode with uniform diffusional access to its surface to which the transport limited current is given by Szabo et al.[9]:

$I_{lim} = (2\pi n F D_0 C_0 l) \times f(\tau)$ Eq.(S2)

with

$$f(\tau) = \frac{2e^{-\sqrt{\pi\tau}/20}}{\sqrt{\pi\tau}} + \frac{1}{\ln[(e^{-\gamma}\tau)^{0.5} + e^{5/3}}$$

$$\tau = \frac{4D_0 t}{r_0^2}$$

where n is the number of electrons transferred, F is the Faraday constant (96485 C mol^{-1}), D_0 is the diffusion coefficient (1.9×10^{-9} $m^2 \cdot s^{-1}$ [10]) for O_2 in 0.1 M KOH (298K), C_0 is the saturated concentration of O_2 in 0.1 M KOH (1.21 mM [10]) and l is the CNT length (20 µm) and r_0 is the CNT radius (15 nm), t is the average step or spike duration time (s), γ = 0.5772156.

Assuming a two-electron reduction process occurring on a single nanotube (n=2), the limiting current (I_{lim}) is derived to be 8.1 nA based on the average duration time (t) of 0.065 s.

References

(1) Inc., N. *Multipul Carbon Nanotubes, Bamboo Structure*. **2017**. https://www.nano-lab.com/bamboopowderd30l520.html.

(2) Banks, C. E.; Crossley, A.; Salter, C.; Wilkins, S. J.; Compton, R. G. Carbon nanotubes contain metal impurities which are responsible for the "electrocatalysis" seen at some nanotube‐modified electrodes. *Angew Chem Int Edit* **2006**, *45* (16), 2533-2537.

(3) Ellison, J.; Batchelor-McAuley, C.; Tschulik, K.; Compton, R. G. The use of cylindrical micro-wire electrodes for nano-impact experiments; facilitating the sub-picomolar detection of single nanoparticles. *Sensors Actuat B-Chem* **2014**, *200*, 47-52.

(4) Li, X.; Lin, C.; Batchelor-McAuley, C.; Laborda, E.; Shao, L.; Compton, R. G. New Insights into Fundamental Electron Transfer from Single Nanoparticle Voltammetry. *J Phys Chem Lett* **2016**, *7* (8), 1554-1558. Li, X.; Batchelor-McAuley, C.; Whitby, S. A.; Tschulik, K.; Shao, L.; Compton, R. G. Single Nanoparticle Voltammetry: Contact Modulation of the Mediated Current. *Angew Chem Int Ed Engl* **2016**, *55* (13), 4296-4299.

(5) Ellison, J.; Tschulik, K.; Stuart, E. J.; Jurkschat, K.; Omanovic, D.; Uhlemann, M.; Crossley, A.; Compton, R. G. Get more out of your data: a new approach to agglomeration and aggregation studies using nanoparticle impact experiments. *ChemistryOpen* **2013**, *2* (2), 69-75.

(6) Lu, Y.; Li, X.; Compton, R. G. Electro-oxidation of amino-functionalized multiwalled carbon nanotubes. *Chem Sci* **2022**, *13* (5), 1355-1366.

(7) Zhang, H.; Lin, C.; Sepunaru, L.; Batchelor-McAuley, C.; Compton, R. G. Oxygen reduction in alkaline solution at glassy carbon surfaces and the role of adsorbed intermediates. *J Electroanal Chem* **2017**, *799*, 53-60.

(8) Guidelli, R.; Compton, R. G.; Feliu, J. M.; Gileadi, E.; Lipkowski, J.; Schmickler, W.; Trasatti, S. Defining the transfer coefficient in electrochemistry: An assessment (IUPAC Technical Report). *Pure Appl Chem* **2014**, *86* (2), 245-258.

(9) Szabo, A.; Cope, D. K.; Tallman, D. E.; Kovach, P. M.; Wightman, R. M. Chronoamperometric current at hemicylinder and band microelectrodes: Theory and experiment. *J Electroanal Chem Interfacial Electrochem* **1987**, *217* (2), 417-423.

(10) Davis, R. E.; Horvath, G. L.; Tobias, C. W. The solubility and diffusion coefficient of oxygen in potassium hydroxide solutions. *Electrochim Acta* **1967**, *12* (3), 287-297.
