# Supplementary material for: Oxygen Reduction Reaction at Single Entity Multiwalled Carbon Nanotubes
Source: J Phys Chem Lett. 2022 Apr 21;13(16):3748–53. doi: 10.1021/acs.jpclett.2c00871 (PMC9059181; doi:10.1021/acs.jpclett.2c00871)
Supplement: Supplementary file 2 — jz2c00871_si_002.pdf [file jz2c00871_si_002.pdf]

jz-2022-00871u.R1

Name: Peer Review Information for "The Oxygen Reduction Reaction at Single Entity Multiwalled Carbon Nanotubes"

First Round of Reviewer Comments

Reviewer: 1

Comments to the Author

The ms by Lu et al studies the oxygen reduction reaction at single multiwalled carbon nanotubes (MWCNTs) by the electrochemical nanoimpact strategy. It also compares the nanoimpact results to the electrochemical response of MWCNTs immobilized on electrodes. The main conclusion is that the ORR reaction is quantified at the single MWCNT level, it is likely related to the quinonoid structures of the MWCNTs (as identified in earlier publications from the group) and these structures are catalytic enough to ensure a near-diffusion limited reduction of O2.

The work is interesting it seems however too specialized to interest the large audience of JPCL. Before any publication different issues should be adressed,

1.Electrochemistry of MWCNTs in N2.

a. The section discusses two potential regions however the E<-0.6V is not shown in figure 1D.it is likely in Fig S11.

b. The caption of Figure 1 should be more explicit: what reductive peakispresented in Figure 1B (highlight in Fig1A? or indicate the potential region incaption).

c. the impact duration between 11 and 78ms. This is not seen in the corresponding SI figure (I rather read 10 to 30ms). Unless it also considers the oxidative impacts.However p4, l34 it is indicated that no impacts were observed at potentials more positive than -0.1V while later, line 61 it is mentionned that anodic impacts are observed at positive potentials. Clarify.

d. the impacts are associated to the quinonoid structure reduction. The charge 1.9pC is used to estimate the number of quinonoid per CNT. The number estimated seems wrong (1 order of magnitude lower than what I estimated). By the way, the projected area of the CNT (in SI) is also wrong.

e. this estimate is also puzzling and should be discussed more carefully. For 1.9pC per CNT (of real area 1.8 10^-8cm2) the surface coverage of the quinone would be 10^-9mol/cm2. It seems amazingly large, I doubt a cnt with so much quinonoid structure would be stable. One could then wonder to what extent the CNT probed by single entity measurments are single objects and not aggregates.

f. In this respect the some authors have used optical microscopy to detect single graphene particles of similar size. It also raises a question concerning how the CNT will approach and attach (or detach) the

microelectrode. I suggest the authors read and quote the recent works from Renault and Dick (ACSNano 2021 and later).

2. Electrochemistry in the presence of O2.

a. The whole mecahanistic approach relies, from the macroscale MWCNT layered assembly electrochemistry in Fig 2, on the over-expression of the first reductive peak in the presence of MWCNTs. This discussion should be re-written (lines 32 to 44 p5).

b. however the single MWCNt detection is not performed on glassy carbon electrode but on a carbon fiber miwrowire. I wonder if the discussion of the macroscale electrochemistry holds. I would have appreciated seeing in SI the CV of the ORR at the C microwire. Definitely O2 is reduced (at diffusion limited rate) at the microwire based on the steady-state -0.4uA detected in Figure 3A. Is it correct?

c. If O2 is reduced at the microwire under diffusion control. I would see that O2 is then depleted at the electrode surface and wonder to what extent O2 could be reduced at a higher diffusional rate at the CNT. Again the work of Renault and Dick would be of itnerest.

d. there is not enough examples of CNT nanoimpact transients documented in the work (provide more examples in SI). If one assumes the insert of Figure 3A is a representative nanoimpact transient, how is estiamted the current presented in Fig 3B? I would read a current between 4 an 10nA at -0.6V which is not the value reported (4+/-0.5nA). This part lacks of proof of reproducibility.

e. I also wonder if the frequency of impacts detected is consistent with the content of CNTs. could the authors comment on that?

Reviewer: 2

Comments to the Author

This is an excellent article from leading group in the field of impact electrochemistry. I have only minor comments:

Figure 2B the inset is too small, and the fonts are too small. The authors may consider stating the parameters of the fits in the text, not in the figure.

The references as:

Impact Electrochemistry of Layered Transition Metal Dichalcogenides

ACS Nano 2015, 9(8), 8474-8483, DOI: 10.1021/acsnano.5b03357

Impact Electrochemistry: Measuring Individual Nanoparticles

ACS Nano 2014, 8(8), 7555-7558, DOI: 10.1021/nn503831r

can be considered to be included in the literature.

Author's Response to Peer Review Comments:

Manuscript ID: jz-2022-00871u

Title: "**The Oxygen Reduction Reaction at Single Entity Multiwalled Carbon Nanotubes**"

Dear Editor and Referees,

Thank you for your careful evaluation of our work submitted to '***The Journal of Physical Chemistry Letters*'.** We much appreciate your considered advice and positive views of the work. We have revised the article and responded fully to all of the referee's comments. A detailed list of corrections and modifications is given below and are highlighted in the manuscript.

We hope that you will find the revised manuscript suitable for publication.

Best regards,

Prof. Richard Compton

Response to Referees

We thank both reviewers for their constructive evaluation and helpful comments on our article. We have modified the text taking account into the reviewers' suggestions. Point by point responses to all of the reviewers' comments are given below.

Reviewer: 1

Comments:

The MS by Lu et al studies the oxygen reduction reaction at single multiwalled carbon nanotubes (MWCNTs) by the electrochemical nanoimpact strategy. It also compares the nanoimpact results to the electrochemical response of MWCNTs immobilized on electrodes. The main conclusion is that the ORR reaction is quantified at the single MWCNT level, it is likely related to the quinonoid structures of the MWCNTs (as identified in earlier publications from the group) and these structures are catalytic enough to ensure a near-diffusion limited reduction of O_2.

The work is interesting it seems however too specialized to interest the large audience of JPCL.

Reply:

We agree that the work is interesting but disagree that it is too specialized. The general readership of JPCL will be interested in the link between the single entity and the ensemble responses, an issue at the heart of current Physical Chemistry, whilst the energy scientists and electrochemists will be interested because of the importance of the ORR in energy devices and the widespread use of CNTs in electrocatalysis.

Before any publication, different issues should be addressed

Reply:

We thank the reviewer for the helpful and constructive comments. In the revised text. we have further carefully checked the manuscript and made the corrections suggested. Responses to specific questions are as follows:

1. Electrochemistry of MWCNTs in N_2.

a. The section discusses two potential regions however the E<-0.6V is not shown in figure 1D.it is likely in Fig S11.

Reply: Thanks for the comment. We apologize for not showing the impact dependency over the whole studied potential region from 0 V to -1.0 V in Figure.1(D). Figure.1(D) has been updated in the main text.

<u>Original:</u>

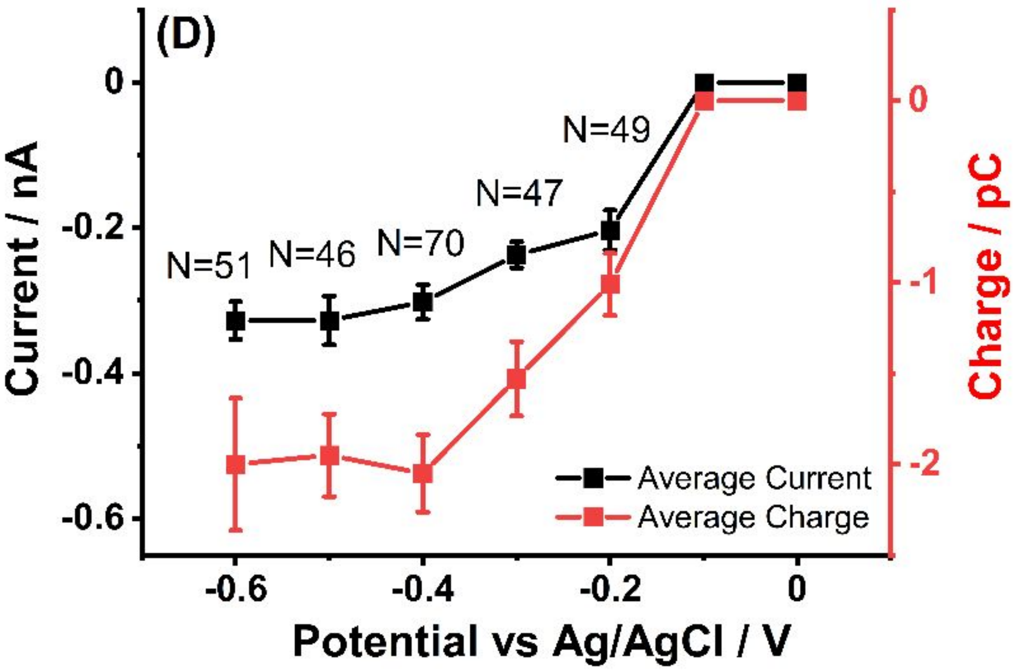

- *Figure.1 (D) Plot of average impact current (black dots) and impact average charge (red dots) as a function of potential from 0 to -1.0 V (The error bars represent the average of at least 40 separate impacts at a carbon microwire electrode for each potential).*

<u>Revised:</u>

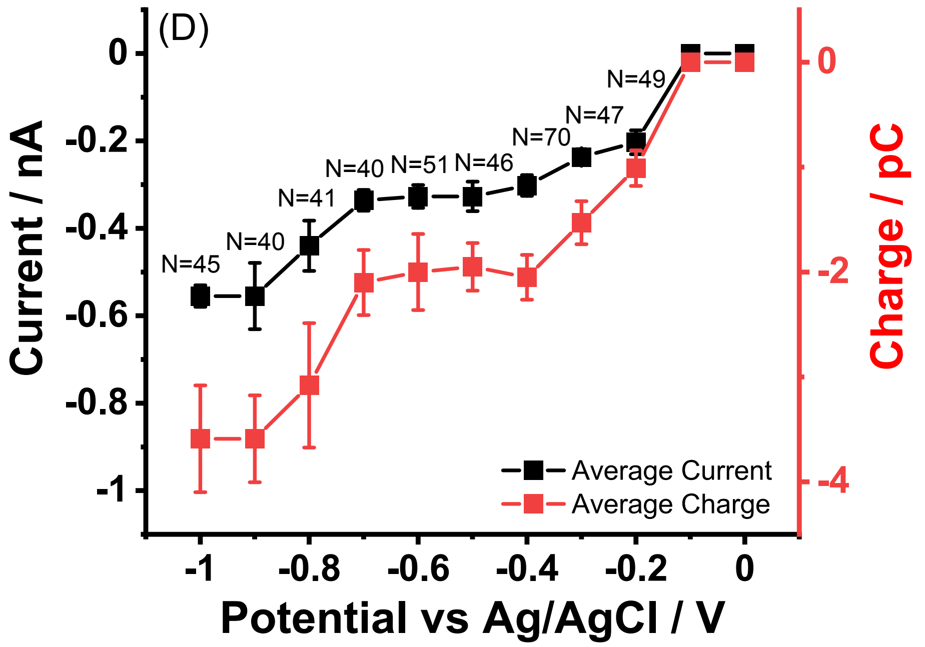

-Figure.1 (D) Plot of average impact current (black dots) and impact average charge (red dots) as a function of potential from 0 to -1.0 V (The error bars represent the average of at least 40 separate impacts at a carbon microwire electrode for each potential).

b. The caption of Figure 1 should be more explicit: what reductive peaks presented in Figure 1B (highlight in Fig1A? or indicate the potential region in caption).

Reply: Thanks for the suggestion. The reductive peak in Figure.1(A) (red curve in Figure 1(A) relates to a cyclic voltammogram measured at a scan rate of 50 mVs^{-1}) observed at MWCNTs modified electrode and is compared with a blank scan (black line) whilst the data in Figure 1(B) relates to different scan rates from 25 mVs^{-1} to 200 mVs^{-1}. The caption of Figure.1 has been modified.

Original:

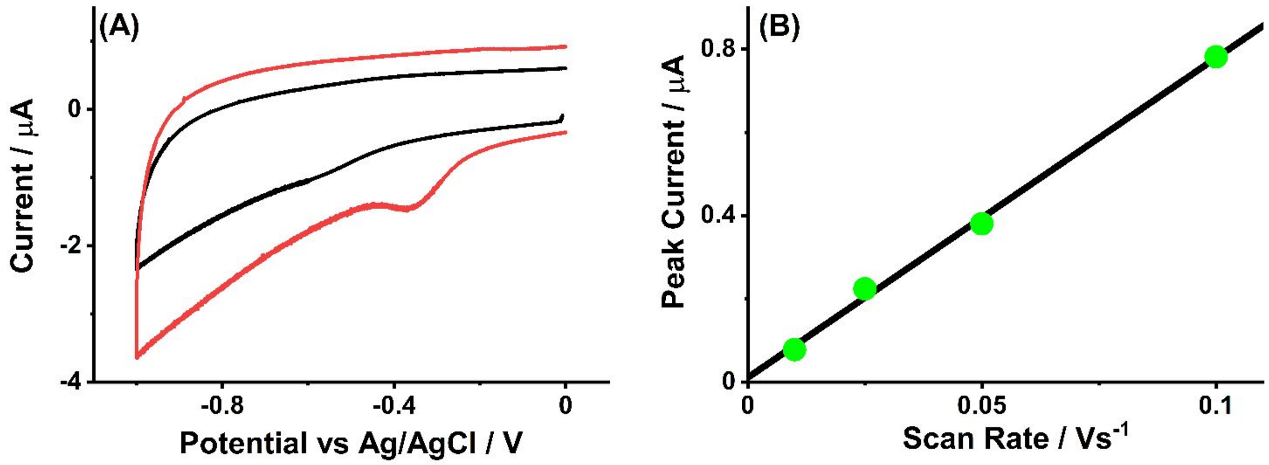

-*Figure.1 (A) A comparison of cyclic voltammograms measured at a bare GCE (black line) of surface area 7.1×10^{-2} cm^2 and the same GCE modified with 0.1 μg MWCNTs (red line) in the potential window from 0 V to -1.0 V at a scan rate of 50 mVs^{-1}; (B) Plot of reductive peak current versus scan rate from 10 mVs^{-1} to 25, 50 and 100 mVs^{-1} after baseline subtraction as shown in Fig.S2);*

Revised:

-*Figure.1 (A) A comparison of cyclic voltammograms measured at a bare GCE (black line) of surface area 7.1×10^{-2} cm^2 and the same GCE modified with 0.1 μg MWCNTs (red line) in the potential window from 0 V to -1.0 V at a scan rate of 50 mVs^{-1}; (B) Plot of reductive peak current observed at a MWCNTs/GCE in the presence of oxygen (red line in A) versus scan rate from 10 mVs^{-1} to 25, 50 and 100 mVs^{-1} after baseline subtraction as shown in Fig.S2);*

c. the impact duration between 11 and 78ms. This is not seen in the corresponding SI figure (I rather read 10 to 30ms). Unless it also considers the oxidative impacts. However, p4, l 34 it is indicated that no impacts were observed at potentials more positive than -0.1V while later, line 61 it is mentioned that anodic impacts are observed at positive potentials. Clarify.

Reply: Thanks for the questions. It should be noted that SI Figure.S7 (A) presented the average duration time of reductive impacts as a function of potential from -1.0 V to -0.2 V. The sentence ‘duration of individual impacts ranging from between 11 and 78 ms’ illustrated in the main text points to the longest (78 ms) and shortest (11 ms) residence time for impact sample respectively (as shown in Figure.1* in the following).

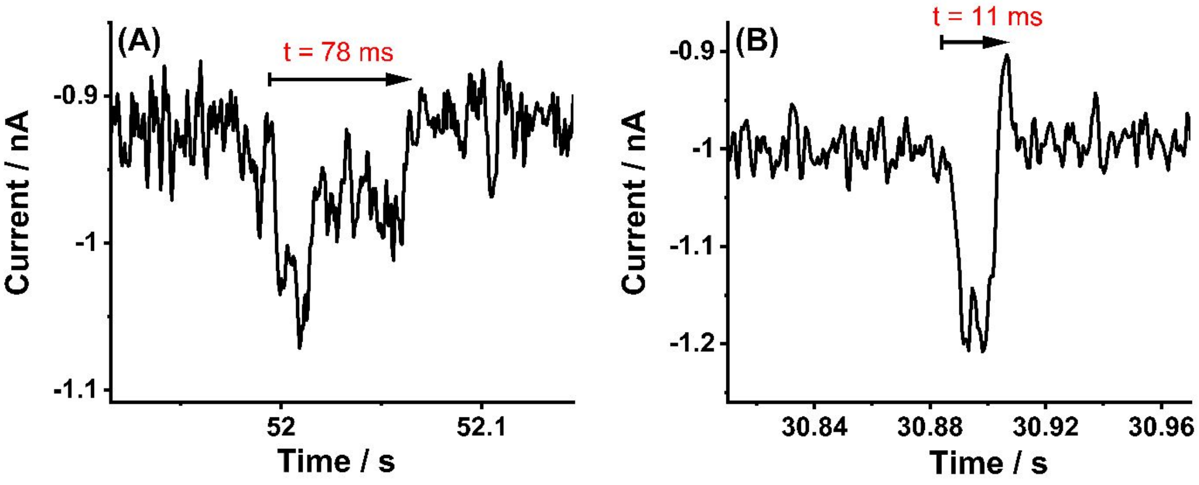

Figure.1 Representative of reductive impact signals with longest (A) or shortest (B) residence time in nitrogen degassed 0.1 M KOH in the presence of 0.001 g L^{-1}MWCNTs applying a fixed potential at -0.4 V vs Ag/AgCl.*

As for the comment that no impacts were observed at potentials more positive than -0.1 V (page 4, line 34) refers to reductive (cathodic) impacts. We have corrected the main text.

Original:

- No impacts were observed for potentials more positive than – 0.1 V or in the absence of CNTs. (Page 4, line 33)

Revised:

- No reductive impacts were observed for potentials more positive than – 0.1 V or in the absence of CNTs.

d. the impacts are associated to the quinonoid structure reduction. The charge 1.9 pC is used to estimate the number of quinonoid per CNT. The number estimated seems wrong (1 order of magnitude lower than what I estimated). By the way, the projected area of the CNT (in SI) is also wrong.

Reply: Thanks for the comment. Please see the corrected calculation as follows:

The external area (S_{mw}) of one single MWCNT

$S_{mw}= \pi d_{MWCNT}\times L=\pi\times 3\times 10^{-8}\ m\times 2.0\times 10^{-5}\ m= 1.9\times 10^{-12}\ m^2$,

where d_{MWCNT} (=30 nm) is the diameter of a carbon tube and L (=20 μm) is the length of each tube.

The number of quinone groups (N_q) introduced into a single MWCNTs particle via electro-reduction can be estimated from nano-impact experiments to be:

$N_q = Q_{MWCNT\text{-}single}/ne = 1.9\times10^{-12}$ C / ($2\times1.6\times10^{-19}$ C) $=6\times10^6$

where $Q_{MWCNT\text{-}single}$ is the average charge (1.9 pC) from the reductive spike current when the potential was between -0.4 V and -0.7 V, n is the number of electrons transferred (n=2, assuming a complete two-electron quinone reduction process here).

Therefore, the coverage (Γ) of quinone groups on a single MWCNTs particle is

$\Gamma = N_q / S_{mw} = 6\times10^6 / 1.9\times10^{-8}$ cm^2 = **3.2×10^{14} molecules per cm^2 of external surface area**

This will be discussed further in the next comment **e**.

As for the projected area of a single CNT, thank you for pointing out this mistake and we have recalculated it. Related estimation of the number of layers of MWCNTs drop-casted on the GC electrodes as follows:

Original:

- The area covered by one MWCNT: $S_{CNT} = 6\times10^{-8}$ cm^2 (SI Section 2)

- The MWCNT layer on the electrode surface was estimated to be at least ca 7 layers, but probably much more (see SI Section 2) from drop-casting 0.1 μg MWCNTs on to a freshly polished GCE. (Main text, Page 3, line 49)

Revised:

- The area covered by one MWCNT: $S_{CNT} = d_{MWCNT}\times L = 3\times10^{-8}$ m$\times2\times10^{-5}$ m= 6×10^{-9} m^2

The modification amount of MWCNTs (2 μL 0.05 gL^{-1}) on the bare GC electrode: $m_{CNT} = 1\times10^{-7}$ g

The number of MWCNTs modified on the surface of modified GCE

$N_{MWCNT}=1\times10^{-7}$ g / 1.3×10^{-14} g = 7.7×10^6 (the mass per carbon tube is 1.3×10^{-14} g [7])

Hence, the total area covered by 7.7×10^6 MWCNTs: $S_{total} = 6\times10^{-9}$ cm^2 $\times$ 7.7×10^6 = 0.046 cm^2

The surface area of GC electrode: $A = (\pi D^2)/4 = 7.1\times10^{-2}$ cm^2

(the diameter of GC electrode is 3.02 ± 0.005 mm)

The number of monolayers of MWCNTs for 2 μL 0.05 gL^{-1} MWCNTs drop cast

$N_{layer} = \frac{S_{total}}{A}$ = 0.046 cm^2 / 0.07 cm^2 $\approx$ 0.65 (SI Section 2)

-The MWCNT layer on the electrode surface was estimated to be at least ca 0.7 layers (see SI Section 2), but probably much more from drop-casting 0.1 μg MWCNTs on to a freshly polished GCE.

e. this estimate is also puzzling and should be discussed more carefully. For 1.9 pC per CNT (of real area 1.8×10^{-8} cm^{2}) the surface coverage of the quinone would be 10^{-9} mol/cm^{2}. It seems amazingly large, I doubt a CNT with so much quinonoid structure would be stable. One could then wonder to what extent the CNT probed by single entity measurements are single objects and not aggregates.

Reply: Thanks for the comments, in the last comment **d**, we have recalculated the surface coverage of the quinone (~5×10^{-10} mol cm^{2}) at a single CNT particle. This corresponds to ca 1 in 6 of the C_6 hexagons on the tube surface becoming quinone groups which is probably an unrealistically high fraction. We therefore conclude that at least some of the impacts may relate to small aggregates of MWCNTs although previous work has shown that in strong electrolyte 'zero-aggregation' collisions were realized [1-3]. The text has been changed as follows :

Original:

- The magnitude of the charge passed in the reductive impacts are consistent with 7×10^{13} molecules per cm^{2} of CNT surface assuming a one-electron process. (Page 4, line 36)

Revised:

- The magnitude of the charge passed in the reductive impacts are consistent with 3.2×10^{14} molecules per cm^{2} of CNT external surface assuming a two-electron process (SI Section 4), which corresponds to an unrealistically high fraction (1 in 6 for quinone groups to C_6 hexagons on the tube surface) suggesting the possibility that some aggregates of MWCNTs are involved in the collision process.

SI Section 4: Estimation of the surface coverage quinone at a single CNT

Here, we compare the number of quinones with the number of hexagonal C_6 rings on the external surface of the MWCNTs:

The external area (S_{mw}) of one single MWCNT

$S_{mw}= \pi d_{MWCNT}\times L=\pi\times 3\times 10^{-8}\ m\times 2.0\times 10^{-5}\ m= 1.9\times 10^{-12}\ m^2$,

where d_{MWCNT} (=30 nm) is the diameter of a carbon tube and L (=20 μm) is the length of each tube.

The number of quinone groups (N_q) introduced into a single MWCNTs particle via electro-reduction can be estimated from nano-impact experiments to be

$N_q = Q_{MWCNT\text{-}single}/ne = 1.9\times10^{-12}$ C / ($2\times1.6\times10^{-19}$ C) $=6\times10^{6}$

where $Q_{MWCNT\text{-}single}$ is the average charge from the reductive spike current when the potential was between -0.4 V and -0.7 V, n is the number of electrons transferred (n=2, assuming a complete two-electron quinone reduction process here).

Therefore, the coverage (Γ) of quinone groups on a single MWCNTs particle is

$\Gamma = N_q / S_{mw} = 6\times10^{6} / 1.9\times10^{-8}$ cm^2 = **3.2×10^{14} molecules per cm^2 of external surface area**

The area (S_h) of one C_6 hexagon composed of six carbon atoms is

$S_h = 3\ d_{c\text{-}c}^2 \times \frac{\sqrt{3}}{2} = 5.2\times10^{-20}$ m^2, where $d_{c\text{-}c}$ (= 0.1421 nm) is the length of the C-C bond

The number of hexagons (N_h) on the external surface of a single MWCNTs particle is

$N_h = S_{mw} / S_h = 1.9\times10^{-12}$ m^2 / 5.2×10^{-20} m^2 = 3.6×10^{7}

The amount ratio between quinone groups and hexagons is N_q / N_h = 1:6.

f. In this respect some authors have used optical microscopy to detect single graphene particles of similar size. It also raises a question concerning how the CNT will approach and attach (or detach) the microelectrode. I suggest the authors read and quote the recent works from Renault and Dick (ACS Nano 2021 and later).

Reply: Thank you for the suggestions. We have cited the references from Renault and Dick (ACS Nano 2021 & Analytical Chemistry 2021) in the main text, which are very helpful to explain the possible dynamic motions of nanoparticles when in contact with the microelectrode.

Original:

- Anodic impacts were observed at positive potentials as reported in the SI Section 4. (Page 4, line 59)

Revised:

- Anodic impacts were observed at positive potentials as reported in the SI Section 5. Last, we note that the shape of individual impact transient has been used to infer the possible collision dynamics in

the case of graphene nanoplatelets [21]. This may also be possible in the case of MWCNTs but is beyond the scope of this letter.

(21) Pendergast, A. D.; Deng, Z.; Maroun, F.; Renault, C.; Dick, J. E. Revealing Dynamic Rotation of Single Graphene Nanoplatelets on Electrified Microinterfaces. *ACS Nano* **2021**, *15* (1), 1250-1258. Pendergast, A. D.; Renault, C.; Dick, J. E. Correlated Optical–Electrochemical Measurements Reveal Bidirectional Current Steps for Graphene Nanoplatelet Collisions at Ultramicroelectrodes. *Anal Chem* **2021**, 93 (5), 2898-2906.

2. Electrochemistry in the presence of O_2.

a. The whole mechanistic approach relies, from the macroscale MWCNT layered assembly electrochemistry in Fig 2, on the over-expression of the first reductive peak in the presence of MWCNTs. This discussion should be re-written (lines 32 to 44 p5).

Reply: Thanks for the suggestion. The discussion in this part has been modified in the main text. Please see the following:

Original:

- Comparison of the response of the bare and MWCNT modified GCE indicates that $Peak_1$ is enhanced over $Peak_2$ in the presence of the MWCNTs but in terms of peak potentials and voltammetric waveshape from the voltammograms are rather similar except for the increase in $Peak_1$ with CNT coverage. The possible causes for differences include changed electrode kinetics between the glassy carbon and the CNTs, altered numbers of sites for adsorption of intermediates, changed diffusion to and from the electrode with the porous layer promoting thin-layer like response as reported in refs [35,36], and altered homogeneous chemical reactivity of the superoxide and other species within the porous layer. (Page 5 line 32-44)

Revised:

- Comparison of the response of the bare and MWCNTs modified GCE indicated that the currents are slightly higher for both peaks on the MWCNTs modified GCE but otherwise the voltammograms are rather similar except at higher coverages reflected by the increase in $Peak_1$ (Figure S10). The possible causes for differences include changed electrode kinetics between the glassy carbon and the CNTs, altered numbers of sites for adsorption of intermediates, changed diffusion to and from the electrode

with the porous layer promoting thin-layer like response as reported in refs [23], and altered homogeneous chemical reactivity of the superoxide and other species within the porous layer.

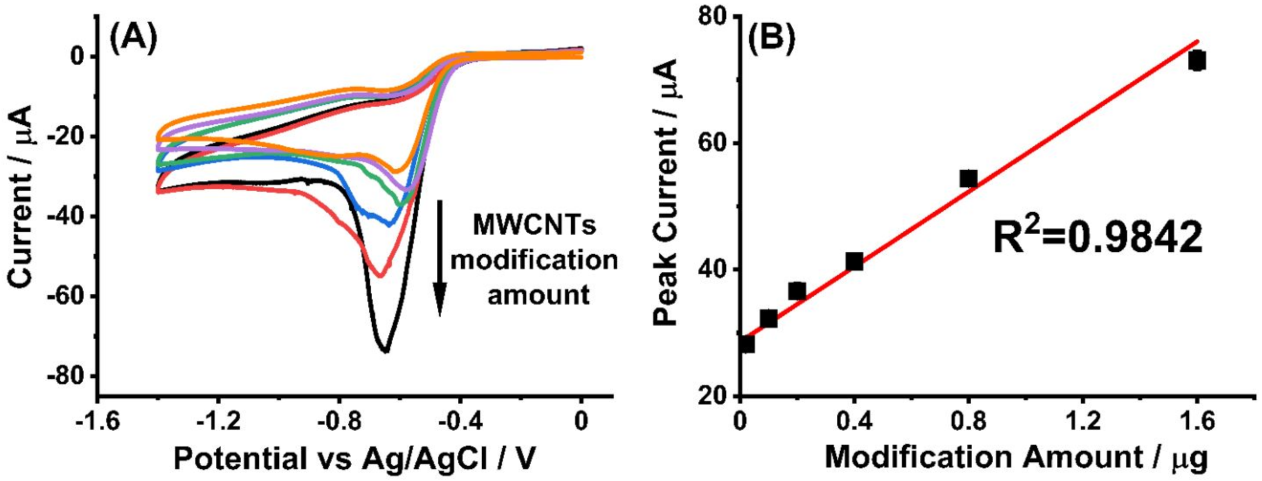

Figure. S10 (A) Cyclic voltammograms of GCE in oxygen saturated 0.1 M KOH solution with different modification amount of MWCNTs from 0.02 to 0.1, 0.2, 0.4, 0.8 and 1.6 µg at a scan rate of 50 mVs^{-1}; (B) The linear relationship between peak current of Peak$_1$ and MWCNTs modification amount.

b. however the single MWCNT detection is not performed on glassy carbon electrode but on a carbon fiber microwire. I wonder if the discussion of the macroscale electrochemistry holds. I would have appreciated seeing in SI the CV of the ORR at the C microwire. Definitely O_2 is reduced (at diffusion limited rate) at the microwire based on the steady-state -0.4 µA detected in Figure 3A. Is it correct?

Reply: Thanks for the comment. Figure S12 below is the cyclic voltammogram at the microwire electrode in the presence of oxygen and has been described in the main text and added into SI Section 9. A steady state current from ORR was observed at the carbon microwire electrode in oxygen saturated 0.1 M KOH solution with a halfwave potential at -0.49 V in the absence of MWCNTs, note that the limiting current magnitude of ca -0.8 µA.

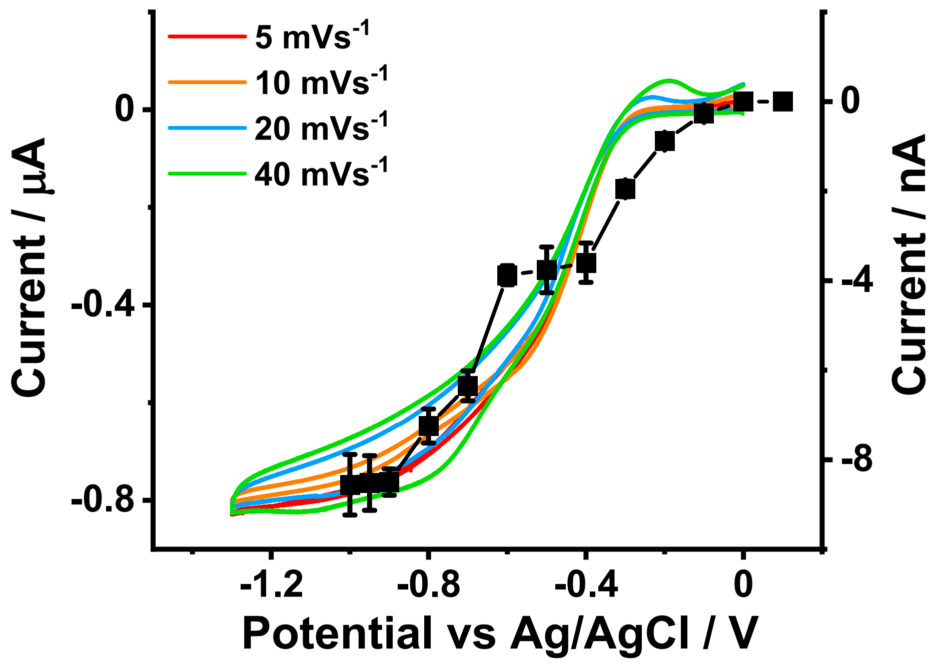

Figure S12 Cyclic voltammograms of carbon microwire electrode in 0.1 M KOH with presence of saturated oxygen in the potential range from 0 V to -1.3 V at different scan rates in comparison with impact currents as a function of potential (orange dots)

<u>Original:</u>

- Clear reductive current steps were seen whilst no impact signals were detected in the absence of MWCNTs (black line in Figure 3(A)). (Page 6, line 12)

<u>Revised:</u>

- Clear reductive current steps were seen whilst no impact signals were detected in the absence of MWCNTs (black line in Figure 3(A)). A steady state ORR reduction wave was observed on the carbon wire electrode in the absence of MWCNTs with a halfwave potential at -0.49 V and a quasi-limiting current at -0.8 µA (Figure S12). The wave is 'drawn out' probably reflecting the merging of the two peaks seen at the GCE. This is discussed further below.

c. If O_2 is reduced at the microwire under diffusion control. I would see that O_2 is then depleted at the electrode surface and wonder to what extent O_2 could be reduced at a higher diffusional rate at the CNT. Again, the work of Renault and Dick would be of interest.

Reply: Thanks for the comments and suggestion. Figure S12 shows that impact signals are seen at potentials less negative than required for the ORR at the microwire electrode. The magnitude of the impact signal is ca two order of magnitude lower than that for the steady ORR signal seen at the

microwire electrode with absence of CNTs. The reason that impact signals are also apparent even at high negative potential superimposed on the underlying ORR current at the microwire electrode is that we suppose the CNT to make electrical contact via its end or slide and since the tube has a larger length than the electrode radius, it extends beyond the diffusion layer near the electrode. Thus, the partial depletion of oxygen at the microwire electrode does not stop impact signals being seen. The related comment has been added in the main text as well.

Original:

- The diffusion controlled limited current for the reduction of oxygen at a single MWCNT tube is estimated to be ~ 8 nA assuming a two-electron process and that the diffusion is to a micro cylindrical electrode with a radius of 15 nm and a length of 20 μm (SI Section 9). (Page 6, line 37)

Revised:

- Figure S12 shows that impact signals are seen at potentials less negative than required for the ORR at the microwire electrode. The magnitude of the impact signal is ca two order of magnitude lower than that for the steady ORR signal seen at the microwire electrode with absence of CNTs. The reason that impact signals are also apparent even at high negative potentials and appear superimposed on the underlying ORR current at the microwire electrode is that we suppose the CNT to make electrical contact via its end or side and since the tube has a larger length than the substrate electrode radius, the CNT extends beyond the diffusion layer near the electrode. Thus, the partial depletion of oxygen at the microwire electrode does not stop impact signals being seen. At the same time distinctive ORR impacts signals are seen at potentials where no current flows on the underlying microwire electrode. The diffusion controlled limited current for the reduction of oxygen at a single MWCNT tube is estimated to be ~ 8 nA assuming a two-electron process and that the diffusion is to a micro cylindrical electrode with a radius of 15 nm and a length of 20 μm (SI Section 12).

d. there is not enough examples of CNT nanoimpact transients documented in the work (provide more examples in SI). If one assumes the insert of Figure 3A is a representative nanoimpact transient, how is estimated the current presented in Fig 3B? I would read a current between 4 an 10nA at -0.6V which is not the value reported (4+/-0.5nA). This part lacks of proof of reproducibility.

Reply: Thanks for the comment, Figure S11 which presents more examples of impact transient (current is between 4 and 10 nA) at -0.6 V has been added to SI Section 8. The average impact current at each potential in Figure 3(B) was estimated from at least 40 impact samples.

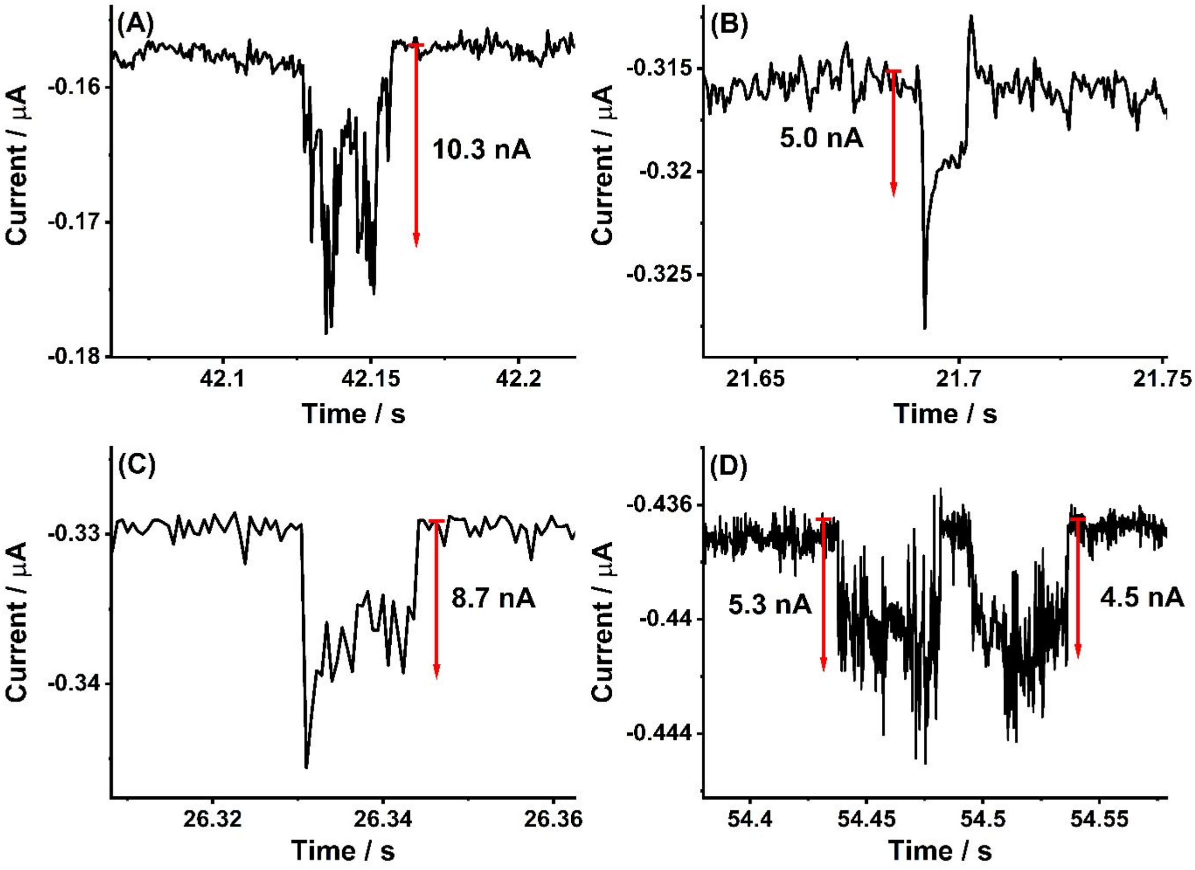

Figure S11 Representative impact signals in oxygen-saturated 0.1 M KOH at -0.6 V vs Ag/AgCl using a carbon wire electrode with presence of 0.001 g L^{-1} MWCNTs.

e. I also wonder if the frequency of impacts detected is consistent with the content of CNTs. could the authors comment on that?

Reply: Thanks for the comment, the frequency of impacts detected at each potential in nitrogen or oxygen saturated solution have been added in the main text as follows:

<u>Original:</u>

- The average impact duration was 22 ± 2 ms with individual impacts ranging from 11 to 78 ms (at least 40 samples for each potential, see Figure S7(A)) and the potential dependency of the average current and charge is shown in Figure 1(D). (Page 4, line 22)
- At the more positive potentials the impacts show a slightly longer average impact time of length 42 ± 13 ms (see Figure S7(B)). (SI Section 5)

- A potential variation study was conducted for the micro-wire electrode at a series of applied potentials from 0.1 V to -1.0 V, Figure 3(B) shows the average current height of the impact features as a function of potential. (Page 6, line 18)

Revised:

- The average impact duration was 22 ± 2 ms with individual impacts ranging from 11 to 78 ms (at least 40 samples for each potential, see Figure S7(A)). The frequency of reductive impact responses maintained a value of 0.068 ± 0.011 s^{-1} over the potential range from -1.0 V to -0.2 V (Figure S8 (A)) and the potential dependency of the average current and charge is shown in Figure 1(D).

- At the more positive potentials the impacts with an average frequency of 0.066 ± 0.015 s^{-1} (Figure S8(B)) show a slightly longer average impact time of length 42 ± 13 ms (see Figure S7(B)).

- The much greater currents flowing when oxygen is present revealed by the reductive impacts with similar average frequency (0.060 ± 0.013 s^{-1}, Figure S14) indicates that the features suggested above to be related to the two one-electron reductions of surface quinone on the CNTs are responsible for catalytic oxygen reduction (Figure S15).

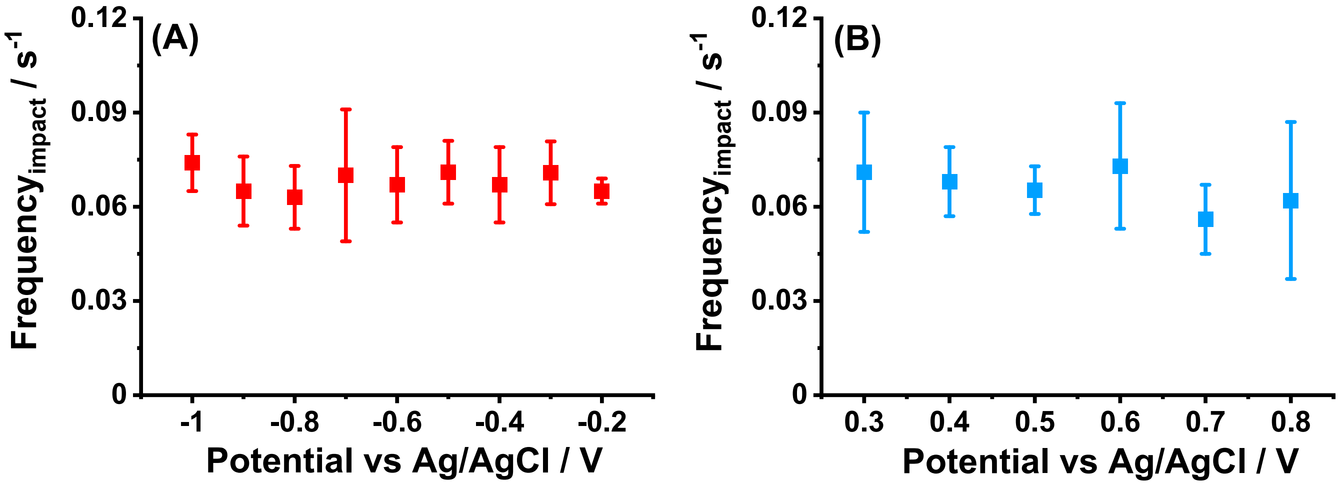

Figure.S8 Impact frequency plot as a function of potential from -0.1 V to -1.0 V (A) and 0.2 V to 0.8 V (B) vs Ag/AgCl in the presence of 0.001 g L^{-1} MWCNTs in nitrogen degassed 0.1 M KOH.

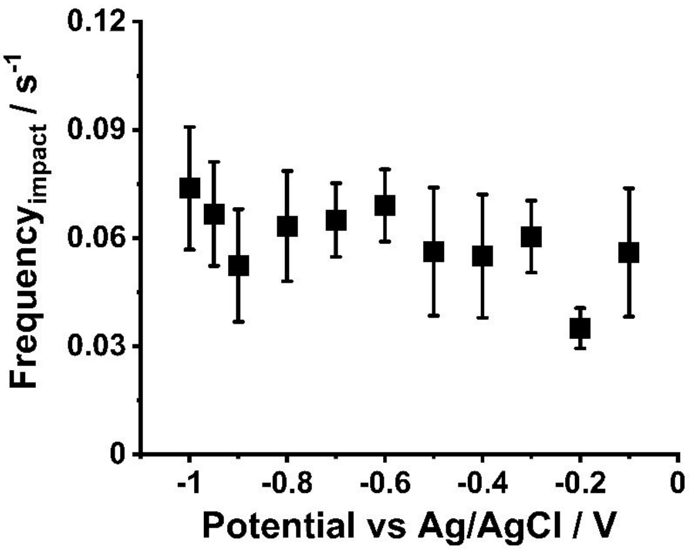

Figure.S14 Impact frequency plot as a function of potential from -0.1 V to -1.0 V vs Ag/AgCl in the presence of 0.001 g L^{-1} MWCNTs in oxygen saturated 0.1 M KOH.

References

(1) Krittayavathananon, A.; Ngamchuea, K.; Li, X.; Batchelor-McAuley, C.; Kätelhön, E.; Chaisiwamongkhol, K.; Sawangphruk, M.; Compton, R. G., Improving single-carbon-nanotube–electrode contacts using molecular electronics. *Journal of Physical Chemistry Letters* **2017,** *8* (16), 3908-3911.

(2) Wang, Y.; Kumar, A. K. S.; Compton, R. G., Optimising Adsorptive Stripping Voltammetry: Strategies and Limitations. *ChemElectroChem* **2021,** *8* (12), 2343-2352.

(3) Kumar, A. K. S.; Miao, R.; Li, D.; Compton, R. G., Do carbon nanotubes catalyse bromine/bromide redox chemistry? *Chemical science* **2021,** *12* (32), 10878-10882.

Reviewer: 2

Comments:

This is an excellent article from leading group in the field of impact electrochemistry. I have only minor comments:

Reply: We thank the reviewer for the positive comments and helpful suggestions. Responses to specific comments are as follows:

a. Figure 2B the inset is too small, and the fonts are too small. The authors may consider stating the parameters of the fits in the text, not in the figure.

Reply: Thanks for the suggestion, we have modified the fonts and inset size in Figure.2(B), and the stated parameters have been moved from the figure to the caption of Figure.2.

Original:

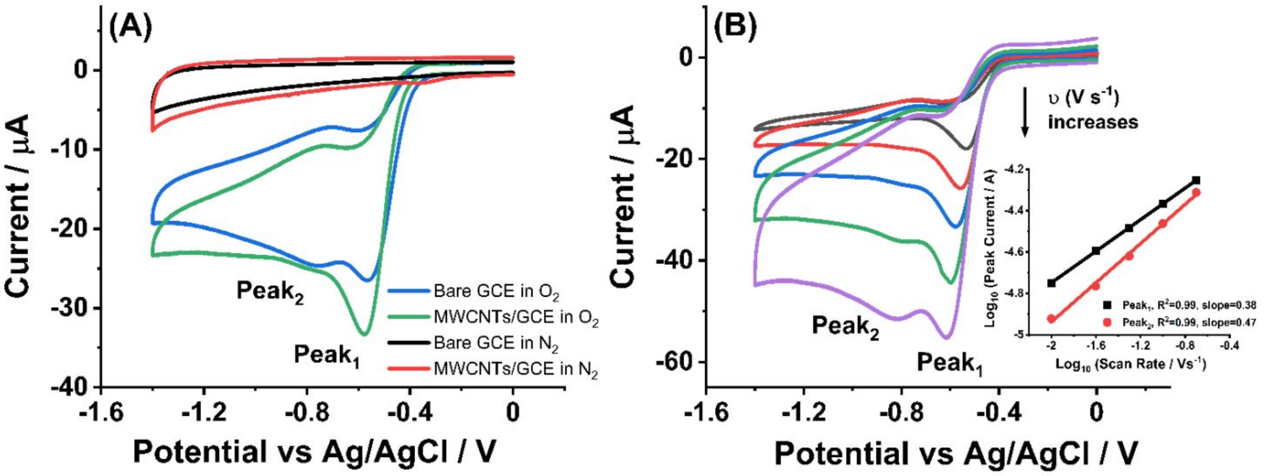

Figure.2 (A) Voltammograms of bare GCE and the same GCE modified with 0.1 μg MWCNTs in 0.1 M KOH in the presence or absence of O_2 at a scan rate of 50 mVs^{-1}; (B) Voltammograms at the 0.1 μg MWCNTs modified GCE in 0.1 M KOH with saturated O_2 as a function of scan rate (Inlay: Plot of peak currents versus scan rates in a log-log form for Peak$_1$ and Peak$_2$).

Revised:

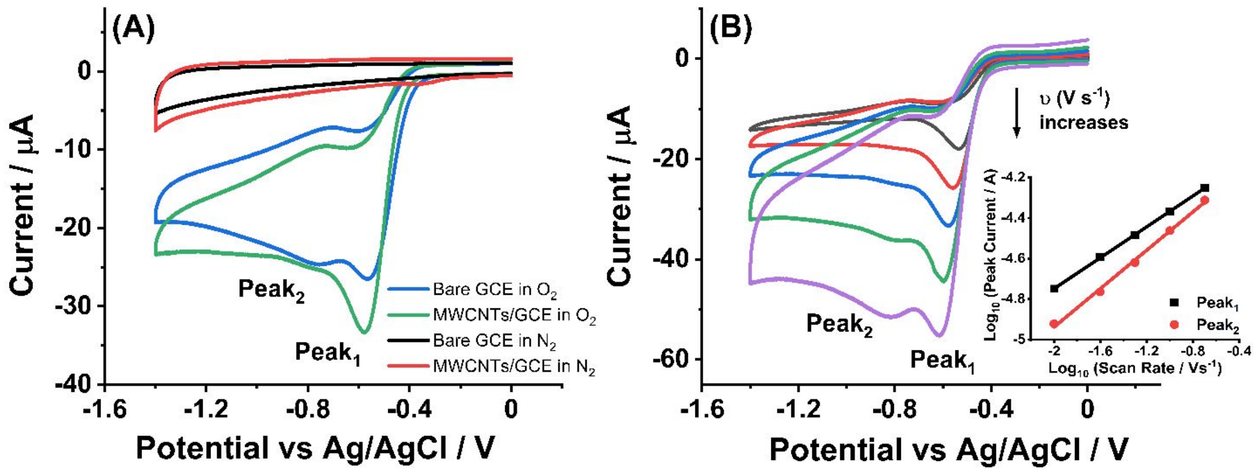

Figure.2 (A) Voltammograms of bare GCE and the same GCE modified with 0.1 μg MWCNTs in 0.1 M KOH in the presence or absence of O_2 at a scan rate of 50 mVs^{-1}; (B) Voltammograms at the 0.1 μg MWCNTs modified GCE in 0.1 M KOH with saturated O_2 as a function of scan rate (Inlay: Plot of peak currents versus scan rates in a log-log form for Peak$_1$ (R^2=0.99, Slope=0.38) and Peak$_2$ (R^2=0.99, Slope=0.47)).

b. The references as follows can be considered to be included in the literature:

Impact Electrochemistry of Layered Transition Metal Dichalcogenides

ACS Nano 2015, 9(8), 8474-8483, DOI: 10.1021/acsnano.5b03357

Impact Electrochemistry: Measuring Individual Nanoparticles

ACS Nano 2014, 8(8), 7555-7558, DOI: 10.1021/nn503831r

Reply: Thanks for the recommendation of two pertinent references. We have cited both of them in the main text of the revision.

Original:

- In the following, we compare the electrocatalytic behavior of MWCNTs for the ORR in aqueous base using ensemble and single entity electrochemistry techniques. (Page 2, line 27)

Revised:

- In the following, we compare the electrocatalytic behavior of MWCNTs for the ORR in aqueous base using ensemble and single entity electrochemistry techniques [12]. (Page 2, line 27)

(12) Pumera, M., Impact electrochemistry: measuring individual nanoparticles. *ACS Nano* **2014,** *8* (8), 7555-7558. Lim, C. S.; Tan, S. M.; Sofer, Z. k.; Pumera, M., Impact electrochemistry of layered transition metal dichalcogenides. *ACS Nano* **2015,** *9* (8), 8474-8483.
